# Supplementary material for: Prevalence and factors associated with exposure to secondhand smoke (SHS) among young people: a cross-sectional study from the Gambia
Source: BMJ Open. 2018 Mar 14;8(3):e019524. doi: 10.1136/bmjopen-2017-019524 (PMC5857680; doi:10.1136/bmjopen-2017-019524)
Supplement: Author's manuscript [file bmjopen-2017-019524.draft_revisions.pdf]

BMJ Open

BMJ Open is committed to open peer review. As part of this commitment we make the peer review history of every article we publish publicly available.

When an article is published we post the peer reviewers' comments and the authors' responses online. We also post the versions of the paper that were used during peer review. These are the versions that the peer review comments apply to.

The versions of the paper that follow are the versions that were submitted during the peer review process. They are not the versions of record or the final published versions. They should not be cited or distributed as the published version of this manuscript.

BMJ Open is an open access journal and the full, final, typeset and author-corrected version of record of the manuscript is available on our site with no access controls, subscription charges or pay-per-view fees (http://bmjopen.bmj.com).

If you have any questions on BMJ Open's open peer review process please email editorial.bmjopen@bmj.com

BMJ Open

Prevalence and determinants of exposure to Second-Hand Smoke (SHS) among young people: a cross-sectional study from The Gambia

Journal:	*BMJ Open*
Manuscript ID	bmjopen-2017-019524
Article Type:	Research
Date Submitted by the Author:	07-Sep-2017
Complete List of Authors:	Jallow, Isatou; UK Center for Tobacco and Alcohol Studies, Division of Epidemiology and Public Health ; Ministry of Health & Social Welfare(MoH&SW), National Public Health Laboratory<br>Britton, John; University of Nottingham, UK Centre for Tobacco and Alcohol Studies,Division of Epidemiology and Public Health<br>Langley, Tessa; University of Nottingham, UK Centre for Tobacco and Alcohol Studies, Division of Epidemiology and Public Health
**Primary Subject Heading**:	Smoking and tobacco
Secondary Subject Heading:	Epidemiology
Keywords:	EPIDEMIOLOGY, PUBLIC HEALTH, Health policy < HEALTH SERVICES ADMINISTRATION & MANAGEMENT

SCHOLARONE™
Manuscripts

only

For peer review only - http://bmjopen.bmj.com/site/about/guidelines.xhtml

Prevalence and determinants of exposure to Second-Hand Smoke (SHS) among young people: a cross-sectional study from The Gambia

Correspondence to: Isatou K. Jallow, UK Centre for Tobacco and Alcohol Studies, Division of Epidemiology and Public Health, University of Nottingham, Clinical Sciences Building 2, City Hospital, Nottingham NG5 1PB, UK and National Public Health Laboratories, Ministry of Health & Social Welfare(MoH&SW); email: jallowisa@hotmail.com and msxikj@nottingham.ac.uk, Telephone +44 (0) 115 82 31945 Ext : 31377.

Professor John Britton: UK Centre for Tobacco and Alcohol Studies, Division of Epidemiology and Public Health, University of Nottingham, Clinical Sciences Building 2, City Hospital, Nottingham, UK

Dr Tessa Langley: UK Centre for Tobacco and Alcohol Studies, Division of Epidemiology and Public Health, University of Nottingham, Clinical Sciences Building 2, City Hospital, Nottingham, UK

Word count: 2555

Key words: Second-hand smoke, exposure, tobacco, students and The Gambia

For peer review only

ABSTRACT

Background Annually 600,000 deaths are attributed to exposure of non-smokers to second-hand smoke (SHS). These include 165,000 among children, about 60% of which occur in Africa and South-East Asia. In 2011 only four countries in the African region had comprehensive smoke-free legislation covering all public places. Given the increasing prevalence of smoking in many low income countries, preventing exposure to SHS is an urgent public health priority, particularly in sub-Sharan Africa.

Objectives The objective of this study was to obtain a reliable and nationally representative estimate of the prevalence of exposure to SHS, and to identify the major risk factors among young people in The Gambia.

Settings and methods We used two-stage cluster random sampling to select students in secondary schools throughout The Gambia, and a self-administered questionnaire to collect data on demographic characteristics and detail indicators on exposure to SHS.

Results Of 10,392 eligible students, 10,289 (99%; 55% girls and 44% boys, age 12-20 years) participated. The proportion of students reporting any exposure to SHS was 97.0%, (enclosed public places 59.2%, outdoor public places 61.4%, school 21..3% and home 38.2%), with majority (96.4%) reporting some exposure outside the home. Exposure to SHS in the home was more common in girls and among older students. Parental education, living with parents and been sent to purchase cigarettes were associated with exposure to SHS both in and outside the home. A majority of students supported public smoking ban. Many were unaware of the harmful effects of exposure to SHS.

Conclusions Exposure to SHS is highly prevalent among students in the Gambia, and occurs mostly outside the home. Interventions to reduce SHS exposure in students are urgently needed.

For peer review only

Strengths and limitation of this study

- This study provides the first comprehensively representative data on the prevalence and determinants of exposure to SHS among young people in The Gambia.
- The participation rate among those sampled was extremely high and UBS and SSS schools were sample from schools throughout the country.
- Self- administered questionnaire were used; students may have under-reported or over-reported their answers.
- The survey was limited to students, it may not represent the smoking prevalence of all young people in this age group

For peer review only

BACKGROUND

Annually six million people die from tobacco use. Of these, an estimated 600,000 deaths are attributed to exposure of non-smokers to second-hand smoke (SHS),(1) including 165,000 deaths among children, of which about 60% occur in Africa and South-East Asia.(2) SHS is associated with diseases such as respiratory and cardiovascular disease, lung cancer and other forms of cancers and accounts for about 1% of the global burden of disease.(3)

In 2004, 40% of children, 33% of male and 35% of female non-smokers were exposed to indoors second hand tobacco smoke worldwide (1) The WHO Framework Convention on Tobacco Control (WHO FCTC) has established that 100% smoke-free environments are the only proven way to adequately protect people from the harmful effects of second-hand tobacco smoke.(4) Even though there has been progress in smoke-free policy adoption, the populations of three-quarters of all countries, including 88% of low-income countries, are vulnerable to the dangers of second-hand smoke through weak or absent smoke-free laws.(5) In 2011 only four countries in the African region had comprehensive smoke-free legislation covering all types of public places.(6) Given the increasing prevalence of smoking in many low income countries, preventing exposure to SHS is an urgent public health priority in these countries, particularly in sub-Sharan Africa.

The Gambia is a West African country of 1.9 million people with a *per capita* GDP of US$471 in 2015.(7) Since 1999, The Gambia has been implementing the Prohibition of Smoking (Public Places) Act 1998, which prohibits tobacco smoking in public places, workplaces, hospitals, public vehicles and in government properties or premises.(8) However, data on the prevalence of exposure to second-hand tobacco smoke and the determinants of SHS are limited. In the Global Youth Tobacco Survey (GYTS), which was conducted in the

Greater Banjul Area in 2008, 45% of students stated that people smoked in their presence at home and 59% were exposed to other people's smoke outside their homes.(9) However these estimates are out of date and the authors are unaware of any studies about the determinants of exposure to SHS among adolescents in The Gambia. To obtain a reliable and nationally representative estimate of the prevalence of exposure to SHS, and to identify the major risk factors among young people in The Gambia, we conducted a survey of SHS prevalence and determinants in a nation-wide sample of Gambian schools.

Methods

Study Population

This study was carried out in a sample of Upper Basic Schools (UBS) and Senior Secondary Schools (SSS) throughout the Gambia, using methods described previously.(10) Nationally representative samples of students in grades 7 to 12 (ages 12 to 20 years) were generated by two-stage cluster sampling: in the first stage, schools were randomly selected from a list of schools with a probability proportional to their enrolment size; and in the second stage, classes within the selected schools were randomly selected from the total number of classes in the schools. All students in the selected classes that attended school on the day of the survey were eligible to participate.

Data Collection and study variables

Participating students completed a self-administered questionnaire collecting data on a range of variables including demographic details, exposure to second hand smoke, support for public smoking regulations and knowledge of the harmful effect of SHS. The questionnaire also included a series of questions on several indicators of tobacco use, smoking susceptibility, exposure to tobacco advertisements and promotion, anti-smoking media

For peer review only

messages, beliefs about the danger of smoking and the perceived benefits of smoking; these data have been reported in a separate publication.(10)

Exposure to SHS was measured in the home, outdoor and enclosed public places, public transportation and school promises; and exposure to SHS was defined as being exposed to SHS on at least one day in the past 7 days in any public place or in the home, or in the past 30 days at school.

The survey was carried out between June and December 2016. The survey was approved by The Gambia Government/Medical Research Council (MRC) Joint Ethics Committee, and by the Ethics Committee of the Faculty of School of Medicine and Health Sciences of the University of Nottingham, UK.

Statistical analysis

Data were analysed in Stata version 14. Proportions and 95% confidence intervals were obtained as estimates of the prevalence of exposure to second-hand smoke. We adjusted associations for *a priori* confounders comprising age, gender and rural/urban area of school, and used multivariate logistic regression analyses to ascertain the predicting factors of SHS exposure.

For peer review only

RESULTS

Characteristics of the study population and prevalence of exposure to SHS

A total of 50 schools throughout the country participated in the study, including 33 Upper Basic and 17 Senior Secondary schools, and 13 private, 27 public and 10 grant-aided schools. A total of 10,395 students were registered in the selected classes, of which 10,289 (99%) students participated in the study. Detailed characteristics of the study participants are summarised in Table 1. Among the total sample, 55.6% were girls and 44.4% were boys. More than half (63.9%) of participants were aged between 14 and 17 years. The majority of the students attended public schools (74.6%), were of Muslim faith (93.1%), lived with their parents (80.2%), lived in homes where smoking was not allowed (70.9%), and had no family members (71.6%) or friends (66.5%) who smoked. About half (43.4%) of the students reported purchasing cigarettes for their parents or others and the majority of the students (97.0%) were exposed to SHS. About 35% of students had seen people smoking inside their school and about a third of those who had seen people smoking in their schools (12.1% of the total sample) had seen teachers smoking.

Participants' place of exposure to SHS

Figure 1 describes the participants' exposure to SHS in different locations. More than half of the students were exposed to SHS for at least one day in the past week in enclosed (59.2%) and outdoor (61.4%) public places, and 38.2 % in the home. The majority (96.4%) of students were exposed to SHS outside the home (enclosed and outdoor public places, public transportation and school) and 95.7 % of the students were exposed to SHS in an enclosed place (enclosed public place, public transportation, school buildings and/or at home). About one in five (21.3%) students had been exposed to SHS at school on at least one day in the previous 30 days.

For peer review only

Table 1 Sociodemographic characteristics of study participants and SHS exposure

Characteristics	Categories	Total (n=10289)	%
**Age group**	12 - 13	960	9.3
	14 - 15	2776	6.9
	16 – 17	3812	37.0
	18 – 19	2221	21.5
	20	525	5.1
**School Type**	UBS	5785	56.2
	SSS	4504	43.7
**School funding**	Public	7678	74.6
	Grant-aided	1052	10.5
	Private	1559	15.1
**School locality**	Rural	2453	23.8
	Urban	7833	76.1
**Religion**	Muslim	9564	93.1
	Christian	602	5.8
	Other	103	1.0
**Living with parents**	Yes	8250	80.2
	No	2029	19.7
**Father's education**	No formal education	2420	23.5
	Primary school	674	6.5
	Secondary school	2120	20.6
	Tertiary	1867	18.1
	Quranic/Arabic school	2097	20.3
	Don`t know	1110	10.7
**Mother's education**	No formal education	3022	29.3
	Primary school	1168	11.3
	Secondary school	2220	21.5
	Tertiary	1024	9.9
	Quranic/Arabic school	1772	17.2
	Don`t know	1080	10.5
**Smoking status**	Non smokers	8565	83.2
	Ever smokers	1719	16.7
**Home smoking rules**	No	7295	70.9
	Sometimes	1085	10.5
	Yes	1906	8.5
**Family smoking**	None	7364	71.6
	Mother	274	2.6
	Father	1199	11.6
	Brother/Sister	718	6.9
	Others	729	7.0
**Number friends who smoke**	None	6790	66.0
	One	673	6.5
	Two	356	3.4
	Three or more	762	7.4
	Not sure	1699	16.5
**Sent to buy cigarettes for parents or others**	No	5816	56.6
	Yes	4459	43.4
**Exposed to SHS**	Exposed	9982	97.0
	Not exposed	304	3.0
**Seen anyone smoking at school**	Yes	3604	35.0
	No	6666	64.9
**Person seen smoking at school**	Friends	807	7.8
	Other students	891	8.6
	Teachers	1232	12.0
	Other staff	674	6.5
	None	6666	64.9

For peer review only

Figure 1 Exposure to SHS at different locations

Frequency of exposure to SHS among study participants

The frequency of SHS exposure at home and school is summarised in Table 2. Approximately 8% of students reported their father smoking in their presence, and 4.7% of students reported their mother smoking in their presence, every day in the past 7 days. Daily exposure to SHS from other family members (11.7%) was much higher compared to exposure from parents. About 4.4% of students were exposed to SHS every day in school buildings or premises.

For peer review only

Table 2 Frequency of exposure to SHS among participants and support for public smoking ban

**Characteristics**	**Total(n=10,289)**	
	**N**	**(%)**
**Exposure to SHS at home**		
**Father**		
About every day	800	7.7
Sometimes	1253	12.1
Never	6265	60.9
Don't have/don't see this person	1958	19.0
**Mother**		
About every day	489	4.7
Sometimes	951	9.2
Never	6834	66.5
Don't have/don't see this person	2000	19.4
**Sibling**		
About every day	468	4.5
Sometimes	1060	10.3
Never	6848	66.6
Don't have/don't see this person	1901	18.5
**Others**		
About every day	1210	11.7
Sometimes	3223	31.3
Never	3802	37.0
Don't have/don't see this person	2309	19.8
**Exposure to SHS at School**		
**Inside school buildings**		
About every day		5.0
Sometimes	516	18.5
Never	1905	52.0
Don't Know	5339	24.1
	2506	
**School premises**		
About every day	459	4.4
Sometimes	1839	17.8
Never	5218	50.7
Don't Know	2762	26.8
**Support for public smoking band and perception of risk of exposure to SHS**		
**Thinks smoking should be banned in enclosed public places**		
Yes	5761	56.0
No	4517	43.9
**Thinks smoking should be banned in outdoor public places**		
Yes	5852	56.9
No	4424	43.0
**Thinks SHS is harmful**		
Definitely not	2736	26.6
Probably not	968	9.4
Probably yes	1296	12.6
Definitely yes	5278	51.3

For peer review only

Support for public smoking ban among and p**erception of risk of exposure to SHS**

Participants' perceptions of the risk of exposure to SHS and support for ban on public smoking ban are also outlined in Table 2. One in four (26.6%) and one in ten (9.4%) participants reported that exposure to SHS was definitely not harmful and probably not harmful respectively. About half of the participants supported a smoking ban in enclosed (56.0%) and outdoor (56.9%) public places

Factors associated with exposure to SHS

The associations between exposure to SHS in the home and outside the home; and socio-demographic characteristics are outlined in Table 3. After adjustment for age, gender and urban or rural location, exposure to SHS was more common among older students aged 18-20 years at home (OR 1.20, 95% CI 1.02 -1.40) and outside the home (OR 1.14, 95% CI 0.83 - 1.56) compare to those aged 12- 14 years. Exposure to SHS at home was significantly more common among girls (OR 1.34, 95% CI 1.22 -1.47), students attending UBS schools (OR 1.40, 95% CI 1.25 -1.57) and grant –aided schools (OR 1.36, 95% CI 1.17 -1.58), Christians (OR 1.22, 95% CI 1.01 -1.47) or other faiths(OR 1.35, 95% CI 0.86 -2.12) compared to Muslims. Students living with their parents (OR 0.83, 95% CI 0.74 -0.93), who were smokers (OR 1.63, 95% CI 1.31 -2.03), had smoking allowed in their homes and had family members or friends who smoked were also significantly more likely to be exposed to SHS at home. In addition, students who were sent to purchase cigarettes (OR 1.98, 95% CI 1.80-2.18) and supported a ban on public smoking were significantly more likely to be exposed to SHS at home. Furthermore students whose mothers had primary education were more likely to be exposed to SHS compared to those having no formal education. Outside the home, lower maternal and higher paternal educational level, living with parents and being sent to purchase cigarettes for others (OR 1.42, 95% CI 1.14 -1.77) were significantly associated with exposure to SHS.

For peer review only

Table 3 Determinants of SHS exposure at home and outside the home among participants

Characteristics	Categories	Total(n=9982)	Home Adjusted OR (95%:CI)	P value	Outside home Adjusted OR (95%:CI)	P value
**Age group**	12 - 14	2184 (96.8)	1	**0.010**	1	**<0.001**
	15 - 17	5129 (97.0)	1.07(0.95-1.21)		1.04(0.80-1.35)	
	18 – 20	2669 (97.1)	1.20(1.02-1.40)		1.14(0.83-1.56)	
**Gender**	Boys	4437 (97.1)	1	**<0.001**	1	0.890
	Girls	5545 (96.1)	1.34(1.22-1.47)		1.01(0.81-1.26)	
**School type**	SSS	4380 (97.2)	1	**<0.001**	1	0.119
	UBS	5602 (96.8)	1.40(1.25-1.57)		0.80(0.61-1.05)	
**School funding**	Public	7464 (97.2)	1	**<0.001**	1	0.121
	Grand –aided	991 (94.2)	1.36(1.17-1.58)		0.59(0.43-0.80)	
	Private	1527 (98.1)	0.80(0.70-0.92)		1.47(1.00-2.17)	
**School locality**	Rural	2389 (97.3)	1	**<0.001**	1	0.360
	Urban	7593 (96.9)	0.71(0.64-0.79)		0.88(0.67-1.15)	
**Religion**	Muslim	9277 (96.9)	1	**0.049**	1	0.346
	Christian	588 (97.6)	1.22(1.01-1.47)		1.16(0.69-1.95)	
	Other	98 (95.1)	1.35(0.86-2.12)		0.56(0.24-1.32)	
**Fathers education**	No education	2326 (96.1)	1	0.150	1	**<0.001**
	Primary	655 (97.1)	0.95(0.78-1.16)		2.01(1.24-3.26)	
	Secondary	2063 (97.3)	1.26(1.09-1.45)		2.36(1.69-3.31)	
	Tertiary	1827 (97.8)	0.88(0.75-1.04)		3.46(2.29-5.22)	
	Quranic/Arabic	2033 (96.9)	0.98(0.85-1.14)		2.49(1.77-3.50)	
	Don`t know	1077 (97.0)	1.14(0.95-1.36)		2.12(1.39-3.23)	
**Mothers education**	No education	2941 (97.3)	1	**<0.001**	1	**<0.001**
	Primary	1137 (97.3)	1.38(1.18-1.61)		0.94(0.62-1.43)	
	Secondary	2176 (98.0)	0.92(0.81-1.04)		0.75(0.52-1.08)	
	Tertiary	1009 (98.5)	0.91(0.77-1.07)		0.41(0.26-0.65)	
	Quranic/Arabic	1676 (94.5)	0.95(0.83-1.08)		0.30(0.21-0.42)	
	Don`t know	1040 (96.2)	0.83(0.71-0.98)		0.45(0.30-0.69)	
**Living with parents**	No	7988 (96.8)	1	**0.001**	1	**0.008**
	Yes	1987 (97.9)	0.83(0.74-0.93)		0.66(0.49-0.89)	
**Smoking Status**	Non smokers	8309 (97.0)	1	**<0.001**	1	0.250
	Ever smokers	1671 (97.2)	1.63(1.31-2.03)		1.46(0.76-2.82)	
**Smoking at home allowed**	No	7060 (96.7)	1	**<0.001**	1	0.524
	Sometimes	1070 (98.6)	2.29(1.99-2.64)		0.88(0.62-1.25)	
	Yes	1849 (97.0)	2.73(2.43-3.07)		0.86(0.65-1.14)	
**Family smoking**	None	7112 (96.5)	1	**<0.001**	1	0.060
	Mother	270 (98.5)	1.66(1.27-2.18)		2.64(0.95-7.29)	
	Father	1177 (98.1)	3.16(2.74-3.65)		0.75(0.54-1.04)	
	Sibling	702 (97.7)	1.76(1.49-2.08)		1.14(0.72-1.81)	
	Others	716 (98.2)	2.09(1.77-2.47)		1.33(0.80-2.21)	
**Number of friends who smoke**	None	6563 (96.6)	1	**<0.001**	1	0.062
	one	653 (97.0)	1.89(1.58-2.26)		1.11(0.71-1.73)	
	Two	344 (96.6)	1.94(1.52-2.47)		1.14(0.63-2.08)	
	Three or more	743(97.5)	1.53(1.28-1.82)		1.39(0.85-2.25)	
	Not sure	1670 (98.2)	1.13(1.00-1.28)		1.63(1.15-2.30)	
**Sent to buy cigarettes**	**No**	5700 (98.0)	1	**<0.001**	1	**0.001**
	**Yes**	4271 (95.7)	1.98(1.80-2.18)		1.42(1.14-1.77)	
**Ban at enclosed public places**	No	5638 (97.8)	1	**<0.001**	1	0.191
	Yes	4336 (95.9)	1.22(1.09-1.37)		1.19(0.91-1.55)	
**Ban at outdoor public places**	No	5713 (97.6)	1	**0.024**	1	0.760
	Yes	4259 (96.2)	0.88(0.78-0.98)		1.04(0.80-1.35)	

For peer review only

DISCUSSION

This is the first study to provide detailed data on exposure to SHS in a nationally representative sample of adolescent school students in The Gambia. We found a very high level of self-reported exposure to SHS among students, and contrary to expectation found that while around two in five respondents reported SHS exposure in the home, a large majority of young people reported exposure in public places. Older students in our sample were generally more likely to be exposed to SHS, as were children under the age of 15. Older students and girls were significantly more like to be exposed to SHS at home compared to boys. Students in our sample were also more likely to be exposed to SHS if their family or friends smoked, if they attended UBS or grant-aided schools, smoking was allowed in the home and among those who were not Muslim. Exposure to SHS at home and outside the home were also associated with parental educational level, though in opposite ways; higher maternal and lowest paternal levels of education were associated with lower exposure. Students who were sent to purchase cigarettes for others were also more likely to be exposed to SHS. Awareness of the harm to health of SHS exposure was low, with more than a quarter of students reporting that exposure was probably or definitely not harmful. However the majority of students supported a smoking ban in both enclosed and outdoor public places.

For logistical reasons we used a self-administered questionnaire for our study, which has been reported to be highly consistent with urinary cotinine level measurement.(11) Our sampling method ensured that the population selected was likely to be highly representative of students attending schools in The Gambia, though we recognise that school participation is far from complete: data from the Ministry of Basic and Secondary Education (MoBSE) indicate gross enrolment rates of 68.12% and 41.2% for UBS and SSS respectively.(12) However, our participation rate among those sampled was extremely high.

For peer review only

Previous studies of smoking and exposure to SHS among students in The Gambia are limited, the most recent and widely quoted being the 2008 GYTS survey. The current study estimated a lower overall prevalence of exposure to SHS than ours, but this could well reflect the restricted local sampling frame used in the GYTS.(9) The high prevalence of exposure to SHS is consistent with earlier studies in the Gambia and other countries in Africa.(13, 14) In the Gambia the Public Smoking Act, which bans smoking in all public places, came into effect in 1998. However, our observation that exposure to SHS remains high and may even have increased since the 2008 GYTS suggests that efforts are still needed to address the enforcement of this law, particularly since public places were the most frequent source of exposure to SHS among young people in The Gambia. Additionally beyond the direct health benefit of smoke-free policies, implementing smoke-free laws, especially in public places, has been shown to change the public acceptance of smoking by the general population.(15, 16) Only 6 of the 47 countries (13%) in the African Region have enacted policies demanding completely smoke-free public places, or else have subnational laws protecting majority of the population.(17) However, compliance with these smoke-free policies varies extensively, and only Seychelles is reported to have achieved a high degree of compliance; most African countries have noted that enforcement of smoke free polices is still a major challenge.(17) Furthermore, similar to previous findings, (18) our study also showed that many students are supportive of polices that ban public smoking; however many are unaware of the harmful effects of exposure to SHS. Adolescent awareness of the harmful health effects of SHS has been shown to be associated with a reduced risk of exposure to SHS,(19, 20) and suggests that improved education on the risks of SHS could lead to reductions in exposure.

We found that older students were more likely to be exposed to SHS both outside the home and inside the home; this is consistent with findings in previous studies among students.(21, 22) Older students have more opportunities to be outside the home in public places where

For peer review only - http://bmjopen.bmj.com/site/about/guidelines.xhtml

smoking is more likely to happen. Our finding that parents' educational level, living with parents and being sent to purchase cigarette for others were significant determinants of exposure to SHS in public places is consistent with previous studies (23-25) and probably arises from the fact that these characteristics all identify contact with others who smoke.

All of the participating schools in this study reported that they had implemented a comprehensive smoke–free campus policy, yet more than a quarter of students reported SHS exposure at school. These findings, which are consistent with previous reports of significant exposure to SHS at school,(26-28) suggest that enforcement of school-based tobacco control measures needs to be strengthened. Studies have shown that in schools with comprehensive policies and high compliance, students are much less likely to report exposure, and report lower intentions to smoke in the future.(29)

Our results showed that parents' educational level, family or friends' smoking status, living with parents, home smoking rules and being sent to purchase cigarette for others were significant determinants of exposure to SHS in the home is consistent with previous studies (23-25). Furthermore it has also been shown that non-smokers exposed to SHS at home are more likely to be susceptible to initiating smoking than those not exposed.(30) These findings emphasize the importance of developing and implementing polices to protect young people from exposure to SHS in private environments like the home. Educating parents about the harmful effects smoking and exposure to SHS could be one of the effect ways to protect young people at home. This will help to protect children, help parents who smoke to quit and discourage others to smoke in their homes.

This study has shown that exposure to SHS is very high among students and that despite smoke-free laws; protection against SHS exposure in public places in the Gambia is still inadequate. There is an urgent need to advocate for interventions to reduce the current level of exposure to SHS and minimise further exposure among students. This underscores the need to develop comprehensive smoke-free laws and strictly enforce these laws in all

environments. Further research is required to determine whether this is a problem among students alone, or reflects a wider pattern of exposure to SHS among the general population.

Acknowledgements: We thank all the students who participated and the teachers for helping in the coordination; and also all the Regional Education Directors and the Ministry of Basic and Secondary Education. We profoundly recognise the contributions of the field workers and staff of the National Public Health Laboratory (NPHL) . We also acknowledged the support of the Tobacco Control Unit of the Ministry of Health and Social Welfare.

Competing interests: None.

Contributors: IJ, study design, data collection, analysis and drafting of manuscript; TL and JB, study design and revision of manuscript.

Funding: This work was supported by scholarship funding from the Islamic Development Bank (IDB) and the UK Centre for Tobacco and Alcohol Studies at the Division Epidemiology and Public Health of the University of Nottingham.

Ethics approval: This research project was reviewed and approved by The Gambia Government/Medical Research Council (MRC) Joint Ethics Committee in The Gambia, and the Research Ethics Committee of the Faculty of Medicine and Health Sciences, University of Nottingham, UK.

Provenance and peer review: externally peer reviewed**.**

Data sharing statement: No additional data is available

For peer review only

REFERENCES

1. Oberg M, Jaakkola MS, Woodward A, et al. Worldwide burden of disease from exposure to second-hand smoke: a retrospective analysis of data from 192 countries. Lancet. 2011;377(9760):139-46.
2. WHO. Worldwide burden of disease from exposure to second-hand smoke. http://wwwwhoint/quantifying_ehimpacts/publications/shsarticle2010/en/ (accessed 20June 2017).
3. Center for Disease Control and Prevention. The Health Consequences of Involuntary Exposure to Tobacco Smoke; A Report of the Surgeon General. 2006.
4. WHO global report on mortality attributed to toobacco. World Health Organization. 2012.
5. WHO Report on the Global Tobacco Epidemic. Raising tax on tobacco; mpower. 2015.
6. WHO Report on the Global Tobacco Epidemic: Warning about the Dangers of Tobacco. 2011.
7. World Bank World Development Indicator Database 2015 http://dataworldbankorg/country/gambia-the _(accessed March 2017).
8. Ministry of Health & Social Welfare (MoHSW). Prohibation of Smoking (Public Places) Act 1998.
9. Manneh E. The Gambia. A Global Youth Tobacco Survey (GYTS) Country Report (2008)2008 08/11/2016. Available from: http://apps.who.int/fctc/reporting/party_reports/gambia_annex2_gyts_2008_final_report.pdf.
10. Jallow IK, Britton J, Langley T. Prevalence and determinants of tobacco use among young people in The Gambia. BMJ Globah Health, in press. 2017.
11. Ekerbicer HC, Celik M, Guler E, et al. Evaluating environmental tobacco smoke exposure in a group of Turkish primary school students and developing intervention methods for prevention. BMC Public Health. 2007;7:202.
12. The Ministry of Basic and Secondary Education & Gambia National Commission for UNESCO. The Gambia National Education For All Review Report 2014. http://unesdoc.unesco.org/images/0023/002314/231425e.pdf.
13. Lee KA, Palipudi KM, English LM, et al. Secondhand smoke exposure and susceptibility to initiating cigarette smoking among never-smoking students in selected African countries: Findings from the Global Youth Tobacco Survey. Prev Med. 2016;91S:S2-S8.
14. Owusu D, Mamudu HM, John RM, et al. Never-Smoking Adolescents' Exposure to Secondhand Smoke in Africa. Am J Prev Med. 2016;51(6):983-98.
15. Husain MJ, English LM, Ramanandraibe N. An overview of tobacco control and prevention policy status in Africa. Prev Med. 2016;91S:S16-S22.
16. Almutairi KM. Prevalence of tobacco use and exposure to environmental tobacco smoke among saudi medical students in Riyadh, Saudi Arabia. J Community Health. 2014;39(4):668-73.
17. Al-Zalabani AH, Amer SM, Kasim KA, et al. Second-Hand Smoking among Intermediate and Secondary School Students in Madinah, Saudi Arabia. Biomed Res Int. 2015;2015:672393.
18. Al-Bedah AM, Qureshi NA, Al-Guhaimani HI, et al. The Global Youth Tobacco Survey - 2007. Comparison with the Global Youth Tobacco Survey 2001-2002 in Saudi Arabia. Saudi Med J. 2010;31(9):1036-43.

For peer review only

19. Rudatsikira E, Siziya S, Dondog J, Muula AS. Prevalence and correlates of environmental tobacco smoke exposure among adolescents in Mongolia. Indian J Pediatr. 2007;74(12):1089-93.
20. Raute LJ, Pednekar MS, Mistry R, Gupta PC, Pimple SA, Shastri SS. Determinants of exposure to second-hand smoke at home and outside the home among students aged 11-17 years: results from the Mumbai Student Tobacco Survey 2010. Indian J Cancer. 2012;49(4):419-24.
21. Rachiotis G, Siziya S, Muula AS, Rudatsikira E, Papastergiou P, Hadjichristodoulou C. Determinants of exposure to Environmental Tobacco Smoke (ETS) among non smoking adolescents (aged 11-17 years old) in Greece: results from the 2004-2005 GYTS Study. Int J Environ Res Public Health. 2010;7(1):284-90.
22. Ordaan ER, Ehrlich RI, Potter P. Environmental tobacco smoke exposure in children: household and community determinants. Arch Environ Health. 1999;54(5):319-27.
23. Peltzer K. Determinants of exposure to second-hand tobacco smoke (SHS) among current non-smoking in-school adolescents (aged 11-18 years) in South Africa: results from the 2008 GYTS study. Int J Environ Res Public Health. 2011;8(9):3553-61.
24. Lee K, Hahn EJ, Riker CA, et al. Secondhand smoke exposure in a rural high school. J Of Sch. Nurs. 2007;23(4):222-8.
25. Cartmell KB, Miner C, Carpenter MJ, et al. Secondhand smoke exposure in young people and parental rules against smoking at home and in the car. Public Health Rep. 2011;126(4):575-82.
26. Gonzales M, Malcoe LH, Kegler MC,et al. Prevalence and predictors of home and automobile smoking bans and child environmental tobacco smoke exposure: a cross-sectional study of U.S.- and Mexico-born Hispanic women with young children. BMC Public Health. 2006;6:265.
27. Fallin A, Roditis M, Glantz SA. Association of campus tobacco policies with secondhand smoke exposure, intention to smoke on campus, and attitudes about outdoor smoking restrictions. Am J Public Health. 2015;105(6):1098-100.
28. Farrelly MC, Evans WN, Sfekas AE. The impact of workplace smoking bans: results from a national survey. Tob Control. 1999;8(3):272-7.
29. Stillman F, Navas-Acien A, Ma J, et al. Second-hand tobacco smoke in public places in urban and rural China. Tob control. 2007;16(4):229-34.
30. Wakefield M, Banham D, Martin J, et al. Restrictions on smoking at home and urinary cotinine levels among children with asthma. Am J Prev Med. 2000;19(3):188-92.
31. Akhtar PC, Haw SJ, Currie DB, Zachary R, et al. Smoking restrictions in the home and secondhand smoke exposure among primary schoolchildren before and after introduction of the Scottish smoke-free legislation. Tob Control. 2009;18(5):409-15.

For peer review only

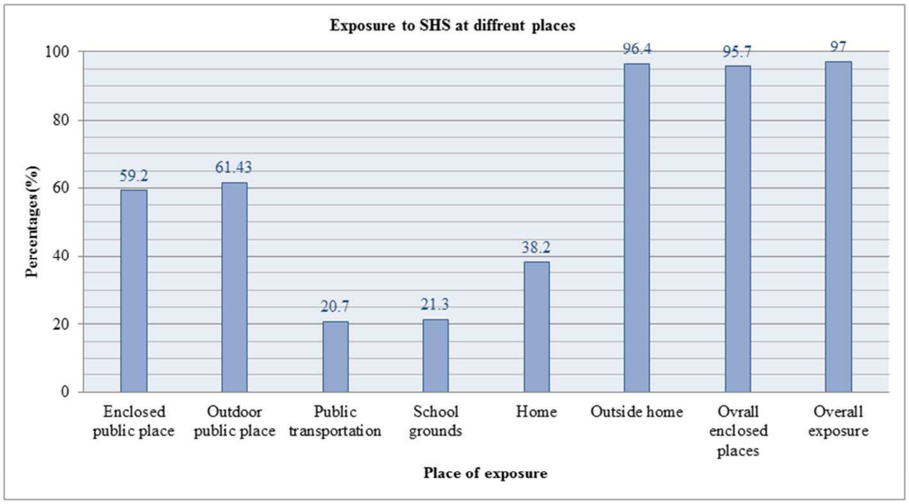

Figure 1 Exposure to SHS at different locations

254x190mm (96 x 96 DPI)

BMJ Open

Prevalence and factors associated with exposure to Second-Hand Smoke (SHS) among young people: a cross-sectional study from The Gambia

Journal:	*BMJ Open*
Manuscript ID	bmjopen-2017-019524.R1
Article Type:	Research
Date Submitted by the Author:	15-Dec-2017

Complete List of Authors:	Jallow, Isatou; UK Center for Tobacco and Alcohol Studies, Division of Epidemiology and Public Health University of Nottingham, Clinical Sciences Building 2,City Hospital; Ministry of Health & Social Welfare(MoH&SW), National Public Health Laboratory<br>Britton, John; University of Nottingham, UK Centre for Tobacco and Alcohol Studies,Division of Epidemiology and Public Health,University of Nottingham, Clinical Sciences Building 2, City Hospital<br>Langley, Tessa; University of Nottingham, UK Centre for Tobacco and Alcohol Studies, Division of Epidemiology and Public Health,University of Nottingham, Clinical Sciences Building 2, City Hospital

**Primary Subject Heading**:	Smoking and tobacco
Secondary Subject Heading:	Epidemiology
Keywords:	EPIDEMIOLOGY, PUBLIC HEALTH, Health policy < HEALTH SERVICES ADMINISTRATION & MANAGEMENT

SCHOLARONE™ Manuscripts

Prevalence and factors associated with exposure to Second-Hand Smoke (SHS) among young people: a cross-sectional study from The Gambia

Correspondence to: Isatou K. Jallow, UK Centre for Tobacco and Alcohol Studies, Division of Epidemiology and Public Health, University of Nottingham, Clinical Sciences Building 2, City Hospital, Nottingham NG5 1PB, UK and National Public Health Laboratories, Ministry of Health & Social Welfare(MoH&SW); email: jallowisa@hotmail.com and msxikj@nottingham.ac.uk, Telephone +44 (0) 115 82 31945 Ext : 31377.

Professor John Britton: UK Centre for Tobacco and Alcohol Studies, Division of Epidemiology and Public Health, University of Nottingham, Clinical Sciences Building 2, City Hospital, Nottingham, UK

Dr Tessa Langley: UK Centre for Tobacco and Alcohol Studies, Division of Epidemiology and Public Health, University of Nottingham, Clinical Sciences Building 2, City Hospital, Nottingham, UK

Word count: 3001

Key words: Second-hand smoke, exposure, tobacco, students and The Gambia

For peer review only

ABSTRACT

Background Annually 600,000 deaths are attributed to exposure of non-smokers to second-hand smoke (SHS). These include 165,000 among children, about 60% of which occur in Africa and South-East Asia. In 2011 only four countries in the African region had comprehensive smoke-free legislation covering all public places. Given the increasing prevalence of smoking in many low income countries, preventing exposure to SHS is an urgent public health priority, particularly in sub-Sharan Africa.

Objectives The objective of this study was to obtain a reliable and nationally representative estimate of the prevalence of exposure to SHS, and to identify the major risk factors among young people in The Gambia.

Settings and methods We used two-stage cluster random sampling to select students in secondary schools throughout The Gambia, and a self-administered questionnaire to collect data on demographic characteristics and detail indicators on exposure to SHS.

Results Of 10,392 eligible students, 10,289 (99%; 55% girls and 44% boys, age 12-20 years) participated. The proportion of students reporting any exposure to SHS was 97.0%, (enclosed public places 59.2%, outdoor public places 61.4%, school 21.3% and home 38.2%), with majority (96.4%) reporting some exposure outside the home. Exposure to SHS in the home was more common in girls and among older students. Parental education, living with parents and been sent to purchase cigarettes were associated with exposure to SHS both in and outside the home. A majority of students supported public smoking ban. Many were unaware of the harmful effects of exposure to SHS.

Conclusions Exposure to SHS is highly prevalent among students in the Gambia, and occurs mostly outside the home. Interventions to reduce SHS exposure in students are urgently needed.

For peer review only - http://bmjopen.bmj.com/site/about/guidelines.xhtml

Strengths and limitation of this study

- This study provides the first comprehensively representative data on the prevalence and determinants of exposure to SHS among young people in The Gambia.
- The participation rate among those sampled was extremely high and UBS and SSS schools were sample from schools throughout the country.
- Self- administered questionnaire were used; students may have under-reported or over-reported their answers.
- The survey was limited to students, it may not represent the smoking prevalence of all young people in this age group

For peer review only

BACKGROUND

Annually six million people die from tobacco use. Of these, an estimated 600,000 deaths are attributed to exposure of non-smokers to second-hand smoke (SHS),(1) including 165,000 deaths among children, of which about 60% occur in Africa and South-East Asia.(2) SHS is associated with diseases such as respiratory and cardiovascular disease, lung cancer and other forms of cancers and accounts for about 1% of the global burden of disease.(3)

In 2004, 40% of children, 33% of male and 35% of female non-smokers were exposed to indoors second hand tobacco smoke worldwide (1) The WHO Framework Convention on Tobacco Control (WHO FCTC) has established that 100% smoke-free environments are the only proven way to adequately protect people from the harmful effects of second-hand tobacco smoke.(4) Even though there has been progress in smoke-free policy adoption, the populations of three-quarters of all countries, including 88% of low-income countries, are vulnerable to the dangers of second-hand smoke through weak or absent smoke-free laws.(5) Currently only seven countries in the African region has comprehensive smoke-free legislation covering all types of public places or at least 90% of the population covered by complete subnational smoke free legislations.(6) Given the increasing prevalence of smoking in many low income countries, preventing exposure to SHS is an urgent public health priority in these countries, particularly in sub-Sharan Africa.

The Gambia is a West African country of 1.9 million people with a *per capita* GDP of US$471 in 2015.(7) Since 1999, The Gambia has been implementing the Prohibition of Smoking (Public Places) Act 1998, which prohibits tobacco smoking in public places, workplaces, hospitals, public vehicles and in government properties or premises.(8) However, data on the prevalence of exposure to second-hand tobacco smoke and the determinants of SHS are limited. In the Global Youth Tobacco Survey (GYTS), which was conducted in the

For peer review only - http://bmjopen.bmj.com/site/about/guidelines.xhtml

Greater Banjul Area in 2008, 45% of students stated that people smoked in their presence at home and 59% were exposed to other people's smoke outside their homes.(9) However these estimates are out of date and the authors are unaware of any studies about the determinants of exposure to SHS among adolescents in The Gambia. To obtain a reliable and nationally representative estimate of the prevalence of exposure to SHS, and to identify the major risk factors among young people in The Gambia, we conducted a survey of SHS prevalence and determinants in a nation-wide sample of Gambian schools.

For peer review only

Methods

Study Population

This study was carried out in a sample of Upper Basic Schools (UBS) and Senior Secondary Schools (SSS) throughout the Gambia: Banjul and Kanifing Municipality which area and the rest of the country, which comprised five regions using methods described previously.(10) In brief, a nationally representative sample of students in UBS (grades 7-9) and SSS (grades 10-12), ages 12 to 20 years were generated by two-stage cluster sampling. In the first stage, a list of all UBS and SSS was obtain from the Ministry of Basic and Secondary Education and schools were randomly selected from the list of schools with a probability proportional to their enrolment size. In the second stage, classes within the selected schools were randomly selected from the total number of classes in the schools. All students in the selected classes that attended school on the day of the survey were eligible to participate. Our study was powered to estimate youth smoking prevalence of 15% with 1% precision, which required a minimum sample size of 4885 (Epi info 7).

Data Collection and study variables

Participating students completed a self-administered questionnaire adapted from the GYTS, collecting data on a range of variables including demographic details, exposure to second

For peer review only - http://bmjopen.bmj.com/site/about/guidelines.xhtml

hand smoke, support for public smoking regulations and knowledge of the harmful effect of SHS. The questionnaire also included a series of questions on several indicators of tobacco use, smoking susceptibility, exposure to tobacco advertisements and promotion, anti-smoking media messages, beliefs about the danger of smoking and the perceived benefits of smoking; these data have been reported in a separate publication. (10)

Self-reported exposure to second hand smoke was the outcome variable and was assessed in the study by the following questions: "During the past 7 days, on how many days has anyone smoked in your presence?: inside your home, in an outdoor public place, in an indoor public place, inside any public transportation"; and "during the past 30 days has anyone smoked in your presence inside the school buildings or premises?" Exposure to SHS was defined as being exposed to SHS on at least one day in the past 7 days in any public place and in the home or in the past 30 days at school. Exposure to SHS outside the home was defined as any exposure at outdoor and indoor public places, inside any public transportation and at school.

The independent variables used in the study were gender, age and religion, school level, school funding type, school locality, parents educational level, tobacco use by family and friends, sent to purchase cigarettes and support for smoke-free bans.

The survey was carried out between June and December 2016. The survey was approved by The Gambia Government/Medical Research Council (MRC) Joint Ethics Committee, and by the Ethics Committee of the Faculty of School of Medicine and Health Sciences of the University of Nottingham, UK.

Statistical analysis

Data were analysed in Stata version 14. Proportions and 95% confidence intervals were obtained as estimates of the prevalence of exposure to second-hand smoke. We adjusted associations for *a priori* confounders comprising age, gender and rural/urban area of school,

For peer review only - http://bmjopen.bmj.com/site/about/guidelines.xhtml

and used multivariate logistic regression analyses to predict factors associated with exposure to SHS.

RESULTS

Characteristics of the study population and prevalence of exposure to SHS

A total of 50 schools throughout the country participated in the study, including 33 Upper Basic and 17 Senior Secondary schools, and 13 private, 27 public and 10 grant-aided schools. A total of 10,395 students were registered in the selected classes, of which 10,289 (99%) students participated in the study. Detailed characteristics of the study participants are summarised in Table 1. Among the total sample, 55.6% were girls and 44.4% were boys. More than half (63.9%) of participants were aged between 14 and 17 years. The majority of the students attended public schools (74.6%), were of Muslim faith (93.1%), lived with their parents (80.2%), lived in homes where smoking was not allowed (70.9%), and had no family members (71.6%) or friends (66.5%) who smoked. About half (43.4%) of the students reported purchasing cigarettes for their parents or others and the majority of the students (97.0%) were exposed to SHS. About 35% of students had seen people smoking inside their school and about a third of those who had seen people smoking in their schools (12.1% of the total sample) had seen teachers smoking.

Participants' place of exposure to SHS

Figure 1 describes the participants' exposure to SHS in different locations. More than half of the students were exposed to SHS for at least one day in the past week in enclosed (59.2%) and outdoor (61.4%) public places, and 38.2 % in the home. The majority (96.4%) of students were exposed to SHS outside the home (enclosed and outdoor public places, public transportation and school) and 95.7 % of the students were exposed to SHS in an enclosed place (enclosed public place, public transportation, school buildings and/or at home). About

For peer review only - http://bmjopen.bmj.com/site/about/guidelines.xhtml

one in five (21.3%) students had been exposed to SHS at school on at least one day in the previous 30 days.

For peer review only

For peer review only - http://bmjopen.bmj.com/site/about/guidelines.xhtml

Table 1 Sociodemographic characteristics of study participants and SHS exposure (N=10289)

Characteristics	Categories	Total (n=10289)	%
**Age group**	12 - 13	960	9.3
	14 - 15	2776	6.9
	16 – 17	3812	37.0
	18 – 19	2221	21.5
	20	525	5.1
**School Type**	UBS	5785	56.2
	SSS	4504	43.7
**School funding**	Public	7678	74.6
	Grant-aided	1052	10.5
	Private	1559	15.1
**School locality**	Rural	2453	23.8
	Urban	7833	76.1
**Religion**	Muslim	9564	93.1
	Christian	602	5.8
	Other	103	1.0
**Living with parents**	Yes	8250	80.2
	No	2029	19.7
**Father's education**	No formal education	2420	23.5
	Primary school	674	6.5
	Secondary school	2120	20.6
	Tertiary	1867	18.1
	Quranic/Arabic school	2097	20.3
	Don`t know	1110	10.7
**Mother's education**	No formal education	3022	29.3
	Primary school	1168	11.3
	Secondary school	2220	21.5
	Tertiary	1024	9.9
	Quranic/Arabic school	1772	17.2
	Don`t know	1080	10.5
**Smoking status**	Non smokers	8565	83.2
	Ever smokers	1719	16.7
**Home smoking rules**	No	7295	70.9
	Sometimes	1085	10.5
	Yes	1906	8.5
**Family smoking**	None	7364	71.6
	Mother	274	2.6
	Father	1199	11.6
	Brother/Sister	718	6.9
	Others	729	7.0
**Number friends who smoke**	None	6790	66.0
	One	673	6.5
	Two	356	3.4
	Three or more	762	7.4
	Not sure	1699	16.5
**Sent to buy cigarettes for parents or others**	No	5816	56.6
	Yes	4459	43.4
**Exposure to SHS**	Exposed	9982	97.0
	Not exposed	304	3.0
**Seen anyone smoking at school**	Yes	3604	35.0
	No	6666	64.9
**Person seen smoking at school**	Friends	807	7.8
	Other students	891	8.6
	Teachers	1232	12.0
	Other staff	674	6.5
	None	6666	64.9

SHS, second-hand smoke
UBS, upper basics schools
SSS, senior secondary schools

For peer review only - http://bmjopen.bmj.com/site/about/guidelines.xhtml

Figure 1 Exposure to SHS at different locations

Frequency of exposure to SHS among study participants

The frequency of SHS exposure at home and school is summarised in Table 2.

Approximately 8% of students reported their father smoking in their presence, and 4.7% of students reported their mother smoking in their presence, every day in the past 7 days. Daily exposure to SHS from other family members (11.7%) was much higher compared to exposure from parents. About 4.4% of students were exposed to SHS every day in school buildings or premises.

For peer review only

For peer review only - http://bmjopen.bmj.com/site/about/guidelines.xhtml

Table 2 Frequency of exposure to SHS among participants and support for public smoking ban (N=10289)

**Characteristics**	**Total(n=10,289) N**	**(%)**
**Exposure to SHS at home**		
**Father**		
About every day	800	7.7
Sometimes	1253	12.1
Never	6265	60.9
Don't have/don't see this person	1958	19.0
**Mother**		
About every day	489	4.7
Sometimes	951	9.2
Never	6834	66.5
Don't have/don't see this person	2000	19.4
**Sibling**		
About every day	468	4.5
Sometimes	1060	10.3
Never	6848	66.6
Don't have/don't see this person	1901	18.5
**Others**		
About every day	1210	11.7
Sometimes	3223	31.3
Never	3802	37.0
Don't have/don't see this person	2309	19.8
**Exposure to SHS at School**		
**Inside school buildings**		
About every day		5.0
Sometimes	516	18.5
Never	1905	52.0
Don't Know	5339	24.1
	2506	
**School premises**		
About every day	459	4.4
Sometimes	1839	17.8
Never	5218	50.7
Don't Know	2762	26.8
**Support for public smoking band and perception of risk of exposure to SHS**		
**Thinks smoking should be banned in enclosed public places**		
Yes	5761	56.0
No	4517	43.9
**Thinks smoking should be banned in outdoor public places**		
Yes	5852	56.9
No	4424	43.0
**Thinks SHS is harmful**		
Definitely not	2736	26.6
Probably not	968	9.4
Probably yes	1296	12.6
Definitely yes	5278	51.3

SHS, second-hand smoke

For peer review only

Support for public smoking ban among and p**erception of risk of exposure to SHS**

Participants' perceptions of the risk of exposure to SHS and support for ban on public smoking ban are also outlined in Table 2. One in four (26.6%) and one in ten (9.4%) participants reported that exposure to SHS was definitely not harmful and probably not harmful respectively. About half of the participants supported a smoking ban in enclosed (56.0%) and outdoor (56.9%) public places

Factors associated with SHS exposure at home and outside the home.

Factors associated with SHS exposure at home

As shown in Table 3, after adjusting for age gender and school location, girls (OR 1.34, 95% CI 1.22 -1.47), students age 18-20 years (OR 1.20, 95% CI 1.02 -1.40), those in UBS schools (OR 1.40, 95% CI 1.25 -1.57) and student attending grant –aided schools (OR 1.36, 95% CI 1.17 -1.58) were significantly more likely to be exposed to SHS. Living with parents (OR 0.83, 95% CI 0.74 -0.93), being a smoker (OR 1.63, 95% CI 1.31 -2.03), having smoking allowed at home and having family members or friends who smoked also significantly increased the risk of students exposure to SHS at home. In addition students who were sent to purchase cigarettes (OR 1.98, 95% CI 1.80-2.18) and supported a ban on public smoking were significantly more likely to be exposed to SHS at home.

Factors associated with SHS exposure outside the home

Outside the home, lower maternal and higher paternal educational level, living with parents and being sent to purchase cigarettes for others (OR 1.42, 95% CI 1.14 -1.77) were significantly associated with increased risk of exposure to SHS (Table 3). In addition older students aged 18-20(OR 1.14, 95% CI 0.83 -1.56) were more likely to be exposed to SHS outside the home compare to younger students age 12-14.

For peer review only - http://bmjopen.bmj.com/site/about/guidelines.xhtml

Table 3 Determinants of SHS exposure at home and outside the home among participants (N=10289)

Characteristics	Categories	Total(n=9982)	Home Adjusted OR (95%:CI)	P value	Outside home Adjusted OR (95%:CI)	P value
**Age group**	12 - 14	2184 (96.8)	1	**0.010**	1	**<0.001**
	15 - 17	5129 (97.0)	1.07(0.95-1.21)		1.04(0.80-1.35)	
	18 – 20	2669 (97.1)	1.20(1.02-1.40)		1.14(0.83-1.56)	
**Gender**	Boys	4437 (97.1)	1	**<0.001**	1	0.890
	Girls	5545 (96.1)	1.34(1.22-1.47)		1.01(0.81-1.26)	
**School type**	SSS	4380 (97.2)	1	**<0.001**	1	0.119
	UBS	5602 (96.8)	1.40(1.25-1.57)		0.80(0.61-1.05)	
**School funding**	Public	7464 (97.2)	1	**<0.001**	1	0.121
	Grand –aided	991 (94.2)	1.36(1.17-1.58)		0.59(0.43-0.80)	
	Private	1527 (98.1)	0.80(0.70-0.92)		1.47(1.00-2.17)	
**School locality**	Rural	2389 (97.3)	1	**<0.001**	1	0.360
	Urban	7593 (96.9)	0.71(0.64-0.79)		0.88(0.67-1.15)	
**Religion**	Muslim	9277 (96.9)	1	**0.049**	1	0.346
	Christian	588 (97.6)	1.22(1.01-1.47)		1.16(0.69-1.95)	
	Other	98 (95.1)	1.35(0.86-2.12)		0.56(0.24-1.32)	
**Fathers education**	No education	2326 (96.1)	1	0.150	1	**<0.001**
	Primary	655 (97.1)	0.95(0.78-1.16)		2.01(1.24-3.26)	
	Secondary	2063 (97.3)	1.26(1.09-1.45)		2.36(1.69-3.31)	
	Tertiary	1827 (97.8)	0.88(0.75-1.04)		3.46(2.29-5.22)	
	Quranic/Arabic	2033 (96.9)	0.98(0.85-1.14)		2.49(1.77-3.50)	
	Don`t know	1077 (97.0)	1.14(0.95-1.36)		2.12(1.39-3.23)	
**Mothers education**	No education	2941 (97.3)	1	**<0.001**	1	**<0.001**
	Primary	1137 (97.3)	1.38(1.18-1.61)		0.94(0.62-1.43)	
	Secondary	2176 (98.0)	0.92(0.81-1.04)		0.75(0.52-1.08)	
	Tertiary	1009 (98.5)	0.91(0.77-1.07)		0.41(0.26-0.65)	
	Quranic/Arabic	1676 (94.5)	0.95(0.83-1.08)		0.30(0.21-0.42)	
	Don`t know	1040 (96.2)	0.83(0.71-0.98)		0.45(0.30-0.69)	
**Living with parents**	No	7988 (96.8)	1	**0.001**	1	**0.008**
	Yes	1987 (97.9)	0.83(0.74-0.93)		0.66(0.49-0.89)	
**Smoking Status**	Non smokers	8309 (97.0)	1	**<0.001**	1	0.250
	Ever smokers	1671 (97.2)	1.63(1.31-2.03)		1.46(0.76-2.82)	
**Smoking at home allowed**	No	7060 (96.7)	1	**<0.001**	1	0.524
	Sometimes	1070 (98.6)	2.29(1.99-2.64)		0.88(0.62-1.25)	
	Yes	1849 (97.0)	2.73(2.43-3.07)		0.86(0.65-1.14)	
**Family smoking**	None	7112 (96.5)	1	**<0.001**	1	0.060
	Mother	270 (98.5)	1.66(1.27-2.18)		2.64(0.95-7.29)	
	Father	1177 (98.1)	3.16(2.74-3.65)		0.75(0.54-1.04)	
	Sibling	702 (97.7)	1.76(1.49-2.08)		1.14(0.72-1.81)	
	Others	716 (98.2)	2.09(1.77-2.47)		1.33(0.80-2.21)	
**Number of friends who smoke**	None	6563 (96.6)	1	**<0.001**	1	0.062
	one	653 (97.0)	1.89(1.58-2.26)		1.11(0.71-1.73)	
	Two	344 (96.6)	1.94(1.52-2.47)		1.14(0.63-2.08)	
	Three or more	743(97.5)	1.53(1.28-1.82)		1.39(0.85-2.25)	
	Not sure	1670 (98.2)	1.13(1.00-1.28)		1.63(1.15-2.30)	
**Sent to buy cigarettes**	**No**	5700 (98.0)	1	**<0.001**	1	**0.001**
	**Yes**	4271 (95.7)	1.98(1.80-2.18)		1.42(1.14-1.77)	
**Ban at enclosed public places**	No	5638 (97.8)	1	**<0.001**	1	0.191
	Yes	4336 (95.9)	1.22(1.09-1.37)		1.19(0.91-1.55)	
**Ban at outdoor public places**	No	5713 (97.6)	1	**0.024**	1	0.760
	Yes	4259 (96.2)	0.88(0.78-0.98)		1.04(0.80-1.35)	

SHS, second-hand smoke
UBS, upper basics schools
SSS, senior secondary schools

DISCUSSION

For peer review only

This is the first study to provide detailed data on exposure to SHS in a nationally representative sample of adolescent school students in The Gambia. We found a very high level of self-reported exposure to SHS among students, and contrary to expectation found that while around two in five respondents reported SHS exposure in the home, a large majority of young people reported exposure in public places. Older students in our sample were generally more likely to be exposed to SHS, as were children under the age of 15. Older students and girls were significantly more like to be exposed to SHS at home compared to boys. Students in our sample were also more likely to be exposed to SHS if their family or friends smoked, if they attended UBS or grant-aided schools, smoking was allowed in the home and among those who were not Muslim. Exposure to SHS at home and outside the home were also associated with parental educational level, though in opposite ways; higher maternal and lowest paternal levels of education were associated with lower exposure. Students who were sent to purchase cigarettes for others were also more likely to be exposed to SHS. Awareness of the harm to health of SHS exposure was low, with more than a quarter of students reporting that exposure was probably or definitely not harmful. However the majority of students supported a smoking ban in both enclosed and outdoor public places.

Our study has some limitations. This was a cross-sectional study and we used a self-administered questionnaire to measure exposure to SHS: students may under or over reported the answers. However students self-reports of exposure to SHS has been reported to be highly consistent with urinary cotinine level measurement.(11) Our sampling method ensured that the population selected was likely to be highly representative of young people in The Gambia, while we recognise that this limits the generalisability of our findings to young people not in school, data from the Ministry of Basic and Secondary Education (MoBSE) indicate gross enrolment rates of 68.1% and 41.2% for UBS and SSS respectively and the universal primary and secondary education initiative which have seen greater number of

For peer review only - http://bmjopen.bmj.com/site/about/guidelines.xhtml

young people go to school in the Gambia .(12) Furthermore the present study has a number of strengths that include a large sample size and high response rate among those interviewed, which also supports the robustness of the study findings. In addition, the study addressed SHS exposure both at home and outside household.

Previous studies of smoking and exposure to SHS among students in The Gambia are limited, the most recent and widely quoted being the 2008 GYTS survey. The current study estimated a lower overall prevalence of exposure to SHS than ours, but this could well reflect the restricted local sampling frame used in the GYTS.(9) The high prevalence of exposure to SHS is consistent with earlier studies in the Gambia and other countries in Africa.(13, 14) In the Gambia the Public Smoking Act, which bans smoking in all public places, came into effect in 1998. However, our observation that exposure to SHS remains high and may even have increased since the 2008 GYTS suggests that efforts are still needed to address the enforcement of this law, particularly since public places were the most frequent source of exposure to SHS among young people in The Gambia. Additionally beyond the direct health benefit of smoke-free policies, implementing smoke-free laws, especially in public places, has been shown to change the public acceptance of smoking by the general population.(15, 16). Most countries in the African region still have weak or even non-existent smoke free laws and compliance with smoke–free laws varies extensively(6). Furthermore enforcement of smoke free polices in most African countries have been identify as a major challenge.(17) Furthermore, similar to previous findings, (18)our study also showed that many students are supportive of polices that ban public smoking; however many are unaware of the harmful effects of exposure to SHS. Adolescent awareness of the harmful health effects of SHS has been shown to be associated with a reduced risk of exposure to SHS,(19, 20) and suggests that improved education on the risks of SHS could lead to reductions in exposure.

For peer review only - http://bmjopen.bmj.com/site/about/guidelines.xhtml

We found that older students were more likely to be exposed to SHS both outside the home and inside the home; this is consistent with findings in previous studies among students.(21, 22) Older students have more opportunities to be outside the home in public places where smoking is more likely to happen. Our finding that parents' educational level, living with parents and being sent to purchase cigarette for others were significant determinants of exposure to SHS in public places is consistent with previous studies (18, 23, 24) and probably arises from the fact that these characteristics all identify contact with others who smoke.

All of the participating schools in this study reported that they had implemented a comprehensive smoke–free campus policy, yet more than a quarter of students reported SHS exposure at school. These findings, which are consistent with previous reports of significant exposure to SHS at school,(25-27) suggest that enforcement of school-based tobacco control measures needs to be strengthened. Studies have shown that in schools with comprehensive policies and high compliance, students are much less likely to report exposure, and report lower intentions to smoke in the future.(28)

Our results showed that parents' educational level, family or friends' smoking status, living with parents, home smoking rules and being sent to purchase cigarette for others were significant determinants of exposure to SHS in the home is consistent with previous studies (18, 21-24). Furthermore it has also been shown that non-smokers exposed to SHS at home are more likely to be susceptible to initiating smoking than those not exposed.(29) Educating parents about the harmful effects smoking and exposure to SHS could be one of the effective ways to protect young people at home. This will help to protect children, help parents who smoke to quit and discourage others to smoke in their homes.

This study has shown that exposure to SHS is very high among students and that despite smoke-free laws; protection against SHS exposure in public places in the Gambia is still inadequate. There is an urgent need to advocate for interventions to reduce the current level

For peer review only - http://bmjopen.bmj.com/site/about/guidelines.xhtml

of exposure to SHS and minimise further exposure among students. This underscores the need to develop comprehensive smoke-free laws and strictly enforce these laws in all environments. Further research is required to determine whether this is a problem among students alone, or reflects a wider pattern of exposure to SHS among the general population.

Acknowledgements: We thank all the students who participated and the teachers for helping in the coordination; and also all the Regional Education Directors and the Ministry of Basic and Secondary Education. We profoundly recognise the contributions of the field workers and staff of the National Public Health Laboratory (NPHL) . We also acknowledged the support of the Tobacco Control Unit of the Ministry of Health and Social Welfare.

Competing interests: None.

Contributors: IJ, study design, data collection, analysis and drafting of manuscript; TL and JB, study design and revision of manuscript.

Funding: This work was supported by the Medical Research Council [grant number MR/K023195/1] through the UK Centre for Tobacco and Alcohol Studies; and a scholarship funding from the Islamic Development Bank (IDB).

Ethics approval: This research project was reviewed and approved by The Gambia Government/Medical Research Council (MRC) Joint Ethics Committee in The Gambia, and the Research Ethics Committee of the Faculty of Medicine and Health Sciences, University of Nottingham, UK.

Provenance and peer review: externally peer reviewed**.**

Data sharing statement: No additional data is available

For peer review only - http://bmjopen.bmj.com/site/about/guidelines.xhtml

REFERENCES

1. Oberg M, Jaakkola MS, Woodward A, Peruga A, Pruss-Ustun A. Worldwide burden of disease from exposure to second-hand smoke: a retrospective analysis of data from 192 countries. Lancet. 2011;377(9760):139-46.
2. WHO. Worldwide burden of disease from exposure to second-hand smoke. http://wwwwhoint/quantifying_ehimpacts/publications/shsarticle2010/en/ (accessed 20June 2017).
3. Center for Disease Control and Prevention. The Health Consequences of Involuntary Exposure to Tobacco Smoke; A Report of the Surgeon General. 2006.
4. WHO. WHO global report on mortality attributed to toobacco. World Health Organization. 2012.
5. WHO Report on the Global Tobacco Epidemic. Raising tax on tobacco; mpower. 2015.
6. WHO Report on the Global Tobacco Epidemic: Monitoring tobacco use and prevention polices. 2017.(accessed December 2017)
7. World Bank World Development Indicator Database 2015 http://dataworldbankorg/country/gambia-the (accessed March 2017).
8. Welfare MoHS. Prohibation of Smoking (Public Places) Act 1998.
9. Manneh E. The Gambia. A Global Youth Tobacco Survey (GYTS) Country Report (2008)2008 08/11/2016. Available from: http://apps.who.int/fctc/reporting/party_reports/gambia_annex2_gyts_2008_final_report.pdf.
10. Jallow IK, Britton J, Langley T. Prevalence and determinants of tobacco use among young people in The Gambia. BMJ Global Health, in press. 2017.
11. Ekerbicer HC, Celik M, Guler E, Davutoglu M, Kilinc M. Evaluating environmental tobacco smoke exposure in a group of Turkish primary school students and developing intervention methods for prevention. BMC Public Health. 2007;7:202.
12. The Ministry of Basic and Secondary Education & Gambia National Commission for UNESCO. The Gambia National Education For All Review Report 2014. http://unesdoc.unesco.org/images/0023/002314/231425e.pdf.
13. Lee KA, Palipudi KM, English LM, Ramanandraibe N, Asma S. Secondhand smoke exposure and susceptibility to initiating cigarette smoking among never-smoking students in selected African countries: Findings from the Global Youth Tobacco Survey. Prev Med. 2016;91S:S2-S8.
14. Owusu D, Mamudu HM, John RM, Ibrahim A, Ouma AE, Veeranki SP. Never-Smoking Adolescents' Exposure to Secondhand Smoke in Africa. Am J Prev Med. 2016;51(6):983-98.
15. Husain MJ, English LM, Ramanandraibe N. An overview of tobacco control and prevention policy status in Africa. Prev Med. 2016;91s:S16-s22.
16. Almutairi KM. Prevalence of tobacco use and exposure to environmental tobacco smoke among saudi medical students in Riyadh, Saudi Arabia. J Community Health. 2014;39(4):668-73.
17. Tumwine J. Implementation of the framework convention on tobacco control in Africa: current status of legislation. Int J Environ Res Public Health. 2011;8(11):4312-31.
18. Peltzer K. Determinants of exposure to second-hand tobacco smoke (SHS) among current non-smoking in-school adolescents (aged 11-18 years) in South Africa: results from the 2008 GYTS study. Int J Environ Res Public Health. 2011;8(9):3553-61.

For peer review only

19. Rudatsikira E, Siziya S, Dondog J, Muula AS. Prevalence and correlates of environmental tobacco smoke exposure among adolescents in Mongolia. Indian J Pediatr. 2007;74(12):1089-93.
20. Raute LJ, Pednekar MS, Mistry R, Gupta PC, Pimple SA, Shastri SS. Determinants of exposure to second-hand smoke at home and outside the home among students aged 11-17 years: results from the Mumbai Student Tobacco Survey 2010. Indian J Cancer. 2012;49(4):419-24.
21. Rachiotis G, Siziya S, Muula AS, Rudatsikira E, Papastergiou P, Hadjichristodoulou C. Determinants of exposure to Environmental Tobacco Smoke (ETS) among non smoking adolescents (aged 11-17 years old) in Greece: results from the 2004-2005 GYTS Study. Int J Environ Res Public Health. 2010;7(1):284-90.
22. Jordaan ER, Ehrlich RI, Potter P. Environmental tobacco smoke exposure in children: household and community determinants. Arch Environ Health. 1999;54(5):319-27.
23. Lee K, Hahn EJ, Riker CA, Hoehne A, White A, Greenwell D, et al. Secondhand smoke exposure in a rural high school. The Journal of School Nursing. 2007;23(4):222-8.
24. Cartmell KB, Miner C, Carpenter MJ, Vitoc CS, Biggers S, Onicescu G, et al. Secondhand smoke exposure in young people and parental rules against smoking at home and in the car. Public Health Rep. 2011;126(4):575-82.
25. Gonzales M, Malcoe LH, Kegler MC, Espinoza J. Prevalence and predictors of home and automobile smoking bans and child environmental tobacco smoke exposure: a cross-sectional study of U.S.- and Mexico-born Hispanic women with young children. BMC Public Health. 2006;6:265.
26. Fallin A, Roditis M, Glantz SA. Association of campus tobacco policies with secondhand smoke exposure, intention to smoke on campus, and attitudes about outdoor smoking restrictions. Am J Public Health. 2015;105(6):1098-100.
27. Farrelly MC, Evans WN, Sfekas AE. The impact of workplace smoking bans: results from a national survey. Tob Control. 1999;8(3):272-7.
28. Stillman F, Navas-Acien A, Ma J, Ma S, Avila-Tang E, Breysse P, et al. Second-hand tobacco smoke in public places in urban and rural China. Tobacco control. 2007;16(4):229-34.
29. Wakefield M, Banham D, Martin J, Ruffin R, McCaul K, Badcock N. Restrictions on smoking at home and urinary cotinine levels among children with asthma. Am J Prev Med. 2000;19(3):188-92.

For peer review only

For peer review only - http://bmjopen.bmj.com/site/about/guidelines.xhtml

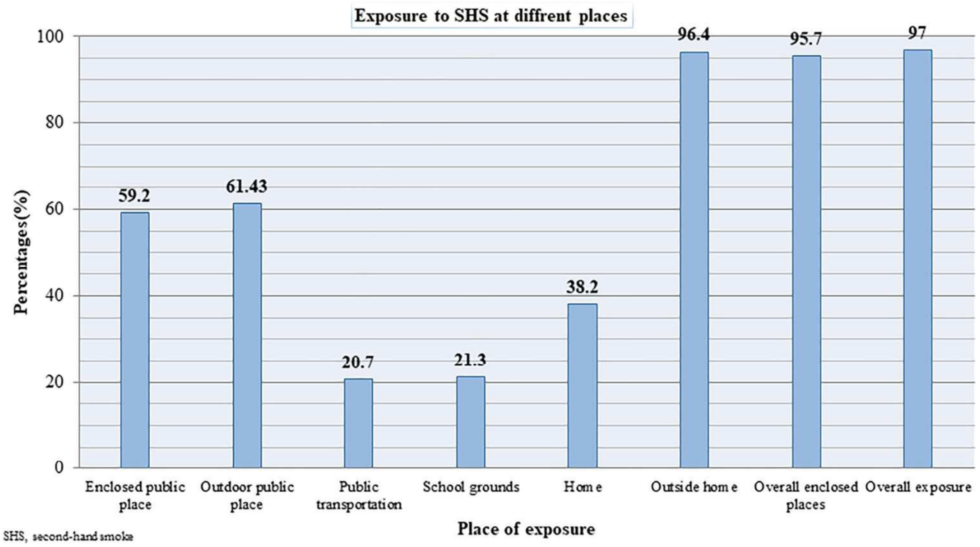

Figure 1 Exposure to SHS at different locations

228x132mm (300 x 300 DPI)

STROBE Statement—Checklist of items that should be included in reports of ***cross-sectional studies***

	Item No	Recommendation	Reported on page #
**Title and abstract**	1	(*a*) Indicate the study's design with a commonly used term in the title or the abstract	1
		(*b*) Provide in the abstract an informative and balanced summary of what was done and what was found	2
**Introduction**			
Background/rationale	2	Explain the scientific background and rationale for the investigation being reported	4
Objectives	3	State specific objectives, including any prespecified hypotheses	4
**Methods**			
Study design	4	Present key elements of study design early in the paper	5
Setting	5	Describe the setting, locations, and relevant dates, including periods of recruitment, exposure, follow-up, and data collection	5
Participants	6	(*a*) Give the eligibility criteria, and the sources and methods of selection of participants	5
Variables	7	Clearly define all outcomes, exposures, predictors, potential confounders, and effect modifiers. Give diagnostic criteria, if applicable	6
Data sources/ measurement	8*	For each variable of interest, give sources of data and details of methods of assessment (measurement). Describe comparability of assessment methods if there is more than one group	6
Bias	9	Describe any efforts to address potential sources of bias	6
Study size	10	Explain how the study size was arrived at	5
Quantitative variables	11	Explain how quantitative variables were handled in the analyses. If applicable, describe which groupings were chosen and why	6
Statistical methods	12	(*a*) Describe all statistical methods, including those used to control for confounding	6
		(*b*) Describe any methods used to examine subgroups and interactions	6
		(*c*) Explain how missing data were addressed	6
		(*d*) If applicable, describe analytical methods taking account of sampling strategy	NA
		(*e*) Describe any sensitivity analyses	NA
**Results**			
Participants	13*	(a) Report numbers of individuals at each stage of study—eg numbers potentially eligible, examined for eligibility, confirmed eligible, included in the study, completing follow-up, and analysed	7
		(b) Give reasons for non-participation at each stage	7
		(c) Consider use of a flow diagram	7
Descriptive data	14*	(a) Give characteristics of study participants (eg demographic, clinical, social) and information on exposures and potential confounders	8
		(b) Indicate number of participants with missing data for each variable of interest	9
Outcome data	15*	Report numbers of outcome events or summary measures	10 & 11
Main results	16	(*a*) Give unadjusted estimates and, if applicable, confounder-adjusted estimates and their precision (eg, 95% confidence interval). Make clear	13

For peer review only

For peer review only - http://bmjopen.bmj.com/site/about/guidelines.xhtml

		which confounders were adjusted for and why they were included	
		(*b*) Report category boundaries when continuous variables were categorized	13
		(*c*) If relevant, consider translating estimates of relative risk into absolute risk for a meaningful time period	NA
Other analyses	17	Report other analyses done—eg analyses of subgroups and interactions, and sensitivity analyses	NA
**Discussion**			
Key results	18	Summarise key results with reference to study objectives	14
Limitations	19	Discuss limitations of the study, taking into account sources of potential bias or imprecision. Discuss both direction and magnitude of any potential bias	14
Interpretation	20	Give a cautious overall interpretation of results considering objectives, limitations, multiplicity of analyses, results from similar studies, and other relevant evidence	15-16
Generalisability	21	Discuss the generalisability (external validity) of the study results	17
**Other information**			
Funding	22	Give the source of funding and the role of the funders for the present study and, if applicable, for the original study on which the present article is based	17

*Give information separately for exposed and unexposed groups.

Note: An Explanation and Elaboration article discusses each checklist item and gives methodological background and published examples of transparent reporting. The STROBE checklist is best used in conjunction with this article (freely available on the Web sites of PLoS Medicine at http://www.plosmedicine.org/, Annals of Internal Medicine at http://www.annals.org/, and Epidemiology at http://www.epidem.com/). Information on the STROBE Initiative is available at www.strobe-statement.org.

For peer review only

BMJ Open

Prevalence and factors associated with exposure to Second-Hand Smoke (SHS) among young people: a cross-sectional study from The Gambia

Journal:	*BMJ Open*
Manuscript ID	bmjopen-2017-019524.R2
Article Type:	Research
Date Submitted by the Author:	29-Jan-2018
Complete List of Authors:	Jallow, Isatou; UK Center for Tobacco and Alcohol Studies, Division of Epidemiology and Public Health University of Nottingham, Clinical Sciences Building 2,City Hospital; Ministry of Health & Social Welfare(MoH&SW), National Public Health Laboratory<br>Britton, John; University of Nottingham, UK Centre for Tobacco and Alcohol Studies,Division of Epidemiology and Public Health,University of Nottingham, Clinical Sciences Building 2, City Hospital<br>Langley, Tessa; University of Nottingham, UK Centre for Tobacco and Alcohol Studies, Division of Epidemiology and Public Health,University of Nottingham, Clinical Sciences Building 2, City Hospital
**Primary Subject Heading**:	Smoking and tobacco
Secondary Subject Heading:	Epidemiology
Keywords:	EPIDEMIOLOGY, PUBLIC HEALTH, Health policy < HEALTH SERVICES ADMINISTRATION & MANAGEMENT

SCHOLARONE™
Manuscripts

Prevalence and factors associated with exposure to Second-Hand Smoke (SHS) among young people: a cross-sectional study from The Gambia

Correspondence to: Isatou K. Jallow, UK Centre for Tobacco and Alcohol Studies, Division of Epidemiology and Public Health, University of Nottingham, Clinical Sciences Building 2, City Hospital, Nottingham NG5 1PB, UK and National Public Health Laboratories, Ministry of Health & Social Welfare (MoH&SW); email: jallowisa@hotmail.com and msxikj@nottingham.ac.uk, Telephone +44 (0) 115 82 31945 Ext : 31377.

Professor John Britton: UK Centre for Tobacco and Alcohol Studies, Division of Epidemiology and Public Health, University of Nottingham, Clinical Sciences Building 2, City Hospital, Nottingham, UK.

Dr Tessa Langley: UK Centre for Tobacco and Alcohol Studies, Division of Epidemiology and Public Health, University of Nottingham, Clinical Sciences Building 2, City Hospital, Nottingham, UK.

Word count: 3001

Key words: Second-hand smoke, exposure, tobacco, students and The Gambia

For peer review only

ABSTRACT

Background: Annually 600,000 deaths are attributed to exposure of non-smokers to second-hand smoke (SHS). These include 165,000 among children, about 60% of which occur in Africa and South-East Asia. In 2011 only four countries in the African region had comprehensive smoke-free legislation covering all public places. Given the increasing prevalence of smoking in many low income countries, preventing exposure to SHS is an urgent public health priority, particularly in sub-Sahara Africa.

Objectives: The objective of this study is to obtain a reliable and nationally representative estimate of the prevalence of exposure to SHS, and to identify the major risk factors among young people in The Gambia.

Settings and methods: We used two-stage cluster random sampling to select students in secondary schools throughout The Gambia, and a self-administered questionnaire to collect data on demographic characteristics and detailed indicators of exposure to SHS.

Results: Of the 10,392 eligible students, 10,289 (99%; 55% girls and 44% boys, age 12-20 years) participated. The proportion of students reporting any exposure to SHS was 97.0%, (enclosed public places 59.2%, outdoor public places 61.4%, school 21.3% and home 38.2%), with 96.4% reporting some exposure outside the home. Exposure to SHS in the home was more common in girls and among older students. Parental education, living with parents and being sent to purchase cigarettes were associated with exposure to SHS both within and outside the home. More than 50% of students supported public smoking ban in both enclosed and outdoor public places. About 35% of students were unaware of the harmful effects of exposure to SHS.

Conclusions: Exposure to SHS is highly prevalent among students in the Gambia, and occurs mostly outside of the home. Interventions to reduce SHS exposure in students are urgently needed.

For peer review only - http://bmjopen.bmj.com/site/about/guidelines.xhtml

Strengths and limitation of this study

- This study provides the first comprehensively representative data on the prevalence and determinants of exposure to SHS among young people in The Gambia.
- The participation rate among those sampled was extremely high and Upper Basic Schools (UBS) and Senior Secondary Schools (SSS) were sampled from schools throughout the Country.
- Self- administered questionnaires were used; students may have under-reported or over-reported their answers.
- The survey was limited to students, it may not represent the smoking prevalence of all young people in this age group

For peer review only

BACKGROUND

Annually six million people die from tobacco use. Of these, an estimated 600,000 deaths are attributed to exposure of non-smokers to second-hand smoke (SHS),(1) including 165,000 deaths among children, of which about 60% occur in Africa and South-East Asia (2). SHS is associated with diseases such as respiratory and cardiovascular disease, lung cancer and other forms of cancers and accounts for about 1% of the global burden of disease (3).

In 2004, 40% of children, 33% male and 35% female non-smokers were exposed to indoors second hand tobacco smoke worldwide (1). The WHO Framework Convention on Tobacco Control (WHO FCTC) has established that 100% smoke-free environments are the only proven way to adequately protect people from the harmful effects of second-hand tobacco smoke (4). Despite the progress made in smoke-free policy adoption, the populations of three-quarters of all countries, including 88% of low-income countries, are vulnerable to the dangers of second-hand smoke due to weak or absent smoke-free laws (5). Currently only seven countries in the African region has comprehensive smoke-free legislation covering all types of public places or at least 90% of the population covered by complete sub-national smoke free legislations (6). Given the increasing prevalence of smoking in many low income countries, preventing exposure to SHS is an urgent public health priority in these countries, particularly in sub-Saharan Africa.

The Gambia is a West African country of 1.9 million people with a *per capita* GDP of US$471 in 2015 (7). Since 1999, The Gambia has been implementing the Prohibition of Smoking (Public Places) Act 1998, which prohibits tobacco smoking in public places, workplaces, hospitals, public vehicles and in government properties or premises (8). However, data on the prevalence of exposure to second-hand tobacco smoke and the determinants of SHS are limited. In the Global Youth Tobacco Survey (GYTS), which was

For peer review only - http://bmjopen.bmj.com/site/about/guidelines.xhtml

conducted in the Greater Banjul Area in 2008, 45% of students stated that people smoked in their presence at home and 59% were exposed to other people's smoke outside their homes (9). However, these estimates are out of date and the authors are not aware of any studies about the determinants of exposure to SHS among adolescents in The Gambia. To obtain a reliable and nationally representative estimate of the prevalence of exposure to SHS, and to identify the major risk factors among young people in The Gambia, we conducted a survey of SHS prevalence and determinants in a nation-wide sample of Gambian schools.

Methods

Study Population

This study was carried out in a sample of Upper Basic Schools (UBS) and Senior Secondary Schools (SSS) throughout the Gambia: Banjul and Kanifing Municipalities and the rest of the country, which comprise five regions using methods described previously (10). In brief, a nationally representative sample of students in UBS (grades 7-9) and SSS (grades 10-12), aged 12 to 20 years were generated by a two-stage cluster sampling. In the first stage, a list of all UBS and SSS was obtain from the Ministry of Basic and Secondary Education and schools were randomly selected from the list of schools with a probability proportional to their enrolment size. In the second stage, classes within the selected schools were randomly selected from the total number of classes in the schools. All students in the selected classes that attended school on the day of the survey were eligible to participate. Our study was powered to estimate youth smoking prevalence of 15% with 1% precision, which required a minimum sample size of 4885 (Epi info 7).

For peer review only

Data Collection and study variables

Participating students completed a self-administered questionnaire adapted from the GYTS, collecting data on a range of variables including demographic details, exposure to second-hand smoke, support for public smoking regulations and knowledge of the harmful effect of SHS. The questionnaire also included series of questions on several indicators of tobacco use, smoking susceptibility, exposure to tobacco advertisements and promotion, anti-smoking media messages, beliefs about the danger of smoking and the perceived benefits of smoking; these data have been reported in a separate publication (10).

Self-reported exposure to second hand smoke was the outcome variable and was assessed in the study by the following questions: "During the past 7 days, on how many days has anyone smoked in your presence?: inside your home, in an outdoor public place, in an indoor public place, inside any public transportation"; and "during the past 30 days has anyone smoked in your presence inside the school buildings or premises?" Exposure to SHS was defined as being exposed to SHS on at least one day in the past 7 days in any public place and in the home or in the past 30 days at school. Exposure to SHS outside the home was defined as any exposure at outdoor and indoor public places, inside any public transportation and at school.

The independent variables used in the study were gender, age and religion, school level, school funding type, school locality, parents' educational level, tobacco use by family and friends, sent to purchase cigarettes and support for smoke-free bans.

The survey was carried out between June and December 2016. The survey was approved by The Gambia Government/Medical Research Council (MRC) Joint Ethics Committee, and by the Ethics Committee of the Faculty of School of Medicine and Health Sciences of the University of Nottingham, UK.

For peer review only - http://bmjopen.bmj.com/site/about/guidelines.xhtml

Statistical analysis

Data were analysed in Stata version 14. Proportions and 95% Confidence Intervals were obtained as estimates of the prevalence of exposure to second-hand smoke. We adjusted associations for a *priori* confounders comprising age, gender and rural/urban area of schools, and used multivariate logistic regression analyses to predict factors associated with exposure to SHS.

RESULTS

Characteristics of the study population and prevalence of exposure to SHS

A total of 50 schools throughout the country participated in the study, including 33 Upper Basic and 17 Senior Secondary schools; comprising of 13 private, 27 public and 10 grant-aided schools. A total of 10,395 students were registered in the selected classes, of which 10,289 (99%) students participated in the study. Detailed characteristics of the study participants are summarised in Table 1. Among the total sample, 55.6% were girls and 44.4% were boys. More than half (63.9%) of participants were aged between 14 and 17 years. The majority (74.6%) of the students attended public schools were of Muslim faith (93.1%), lived with their parents (80.2%), lived in homes where smoking was not allowed (70.9%), and had no family members (71.6%) or friends (66.5%) who smoked. About half (43.4%) of the students reported purchasing cigarettes for their parents or others and 97.0% of students were exposed to SHS. About 35% of students had seen people smoking inside their school and about a third of those who had seen people smoking in their schools (12.1% of the total sample) had seen teachers smoking.

Participants' place of exposure to SHS

Figure 1 describes the participants' exposure to SHS in different locations. More than half of the students were exposed to SHS for at least one day in the past week in enclosed (59.2%) and outdoor (61.4%) public places, and 38.2 % in the home. 96.4% of students were exposed

For peer review only

For peer review only - http://bmjopen.bmj.com/site/about/guidelines.xhtml

to SHS outside the home (enclosed and outdoor public places, public transportation and school) and 95.7% of the students were exposed to SHS in an enclosed place (enclosed public place, public transportation, school buildings and/or at home). About one in five (21.3%) students had been exposed to SHS at school on at least one day in the previous 30 days.

For peer review only

For peer review only - http://bmjopen.bmj.com/site/about/guidelines.xhtml

Table 1 Sociodemographic characteristics of study participants and SHS exposure (N=10289)

Characteristics	Categories	Total (n=10289)	%
**Age group**	12 - 13	960	9.3
	14 - 15	2776	6.9
	16 – 17	3812	37.0
	18 – 19	2221	21.5
	20	525	5.1
**School Type**	UBS	5785	56.2
	SSS	4504	43.7
**School funding**	Public	7678	74.6
	Grant-aided	1052	10.5
	Private	1559	15.1
**School locality**	Rural	2453	23.8
	Urban	7833	76.1
**Religion**	Muslim	9564	93.1
	Christian	602	5.8
	Other	103	1.0
**Living with parents**	Yes	8250	80.2
	No	2029	19.7
**Father's education**	No formal education	2420	23.5
	Primary school	674	6.5
	Secondary school	2120	20.6
	Tertiary	1867	18.1
	Quranic/Arabic school	2097	20.3
	Don`t know	1110	10.7
**Mother's education**	No formal education	3022	29.3
	Primary school	1168	11.3
	Secondary school	2220	21.5
	Tertiary	1024	9.9
	Quranic/Arabic school	1772	17.2
	Don`t know	1080	10.5
**Smoking status**	Non smokers	8565	83.2
	Ever smokers	1719	16.7
**Home smoking rules**	No	7295	70.9
	Sometimes	1085	10.5
	Yes	1906	8.5
**Family smoking**	None	7364	71.6
	Mother	274	2.6
	Father	1199	11.6
	Brother/Sister	718	6.9
	Others	729	7.0
**Number friends who smoke**	None	6790	66.0
	One	673	6.5
	Two	356	3.4
	Three or more	762	7.4
	Not sure	1699	16.5
**Sent to buy cigarettes for parents or others**	No	5816	56.6
	Yes	4459	43.4
**Exposure to SHS**	Exposed	9982	97.0
	Not exposed	304	3.0
**Seen anyone smoking at school**	Yes	3604	35.0
	No	6666	64.9
**Person seen smoking at school**	Friends	807	7.8
	Other students	891	8.6
	Teachers	1232	12.0
	Other staff	674	6.5
	None	6666	64.9

SHS, second-hand smoke
UBS, upper basics schools
SSS, senior secondary schools

For peer review only - http://bmjopen.bmj.com/site/about/guidelines.xhtml

Figure 1 Exposure to SHS at different locations

Frequency of exposure to SHS among study participants

The frequency of SHS exposure at home and school is summarised in Table 2. Approximately 8% of students reported their father smoking in their presence, and 4.7% of students reported their mother smoking in their presence, every day in the past 7days. Daily exposure to SHS from other family members (11.7%) was much higher compared to exposure from parents. About 4.4% of students were exposed to SHS every day in school buildings or premises.

For peer review only

For peer review only - http://bmjopen.bmj.com/site/about/guidelines.xhtml

Table 2 Frequency of exposure to SHS among participants and support for public smoking ban (N=10289)

Characteristics	Total(n=10,289) N	(%)
**Exposure to SHS at home**		
**Father**		
About every day	800	7.7
Sometimes	1253	12.1
Never	6265	60.9
Don't have/don't see this person	1958	19.0
**Mother**		
About every day	489	4.7
Sometimes	951	9.2
Never	6834	66.5
Don't have/don't see this person	2000	19.4
**Sibling**		
About every day	468	4.5
Sometimes	1060	10.3
Never	6848	66.6
Don't have/don't see this person	1901	18.5
**Others**		
About every day	1210	11.7
Sometimes	3223	31.3
Never	3802	37.0
Don't have/don't see this person	2309	19.8
**Exposure to SHS at School**		
**Inside school buildings**		
About every day		5.0
Sometimes	516	18.5
Never	1905	52.0
Don't Know	5339	24.1
	2506	
**School premises**		
About every day	459	4.4
Sometimes	1839	17.8
Never	5218	50.7
Don't Know	2762	26.8
**Support for public smoking band and perception of risk of exposure to SHS**		
**Thinks smoking should be banned in enclosed public places**		
Yes	5761	56.0
No	4517	43.9
**Thinks smoking should be banned in outdoor public places**		
Yes	5852	56.9
No	4424	43.0
**Thinks SHS is harmful**		
Definitely not	2736	26.6
Probably not	968	9.4
Probably yes	1296	12.6
Definitely yes	5278	51.3

SHS, second-hand smoke

For peer review only

For peer review only - http://bmjopen.bmj.com/site/about/guidelines.xhtml

Support for public smoking ban among participants and perception of risk of exposure to SHS

Participants' perceptions of the risk of exposure to SHS and support for ban on public smoking are also outlined in Table 2. One in four (26.6%) and one in ten (9.4%) participants reported that exposure to SHS was definitely not harmful and probably not harmful respectively. About half of the participants supported a smoking ban in enclosed (56.0%) and outdoor (56.9%) public places

Factors associated with SHS exposure at home and outside the home.

Factors associated with SHS exposure at home

As shown in Table 3, after adjusting for age, gender and school location, girls (OR 1.34, 95% CI 1.22 -1.47), students aged 18-20 years (OR 1.20, 95% CI 1.02 -1.40), those in UBS schools (OR 1.40, 95% CI 1.25 -1.57) and student attending grant –aided schools (OR 1.36, 95% CI 1.17 -1.58) were significantly more likely to be exposed to SHS. Living with parents (OR 0.83, 95% CI 0.74 -0.93), being a smoker (OR 1.63, 95% CI 1.31 -2.03), having smoking allowed at home and having family members or friends who smoked also significantly increased the risk of students exposure to SHS at home. In addition, students who were sent to purchase cigarettes (OR 1.98, 95% CI 1.80-2.18) and supported a ban on public smoking were significantly more likely to be exposed to SHS at home.

Factors associated with SHS exposure outside the home

Outside the home, lower maternal and higher paternal educational level, living with parents and being sent to purchase cigarettes for others (OR 1.42, 95% CI 1.14 -1.77) were significantly associated with increased risk of exposure to SHS (Table 3). In addition, older students aged 18-20 (OR 1.14, 95% CI 0.83 -1.56) were more likely to be exposed to SHS outside the home compared to younger students aged 12-14.

For peer review only - http://bmjopen.bmj.com/site/about/guidelines.xhtml

Table 3 Determinants of SHS exposure at home and outside the home among participants (N=10289)

Characteristics	Categories	Total(n=9982)	Home Adjusted OR (95%:CI)	P value	Outside home Adjusted OR (95%:CI)	P value
**Age group**	12 - 14	2184 (96.8)	1	**0.010**	1	**<0.001**
	15 - 17	5129 (97.0)	1.07(0.95-1.21)		1.04(0.80-1.35)	
	18 – 20	2669 (97.1)	1.20(1.02-1.40)		1.14(0.83-1.56)	
**Gender**	Boys	4437 (97.1)	1	**<0.001**	1	0.890
	Girls	5545 (96.1)	1.34(1.22-1.47)		1.01(0.81-1.26)	
**School type**	SSS	4380 (97.2)	1	**<0.001**	1	0.119
	UBS	5602 (96.8)	1.40(1.25-1.57)		0.80(0.61-1.05)	
**School funding**	Public	7464 (97.2)	1	**<0.001**	1	0.121
	Grand –aided	991 (94.2)	1.36(1.17-1.58)		0.59(0.43-0.80)	
	Private	1527 (98.1)	0.80(0.70-0.92)		1.47(1.00-2.17)	
**School locality**	Rural	2389 (97.3)	1	**<0.001**	1	0.360
	Urban	7593 (96.9)	0.71(0.64-0.79)		0.88(0.67-1.15)	
**Religion**	Muslim	9277 (96.9)	1	**0.049**	1	0.346
	Christian	588 (97.6)	1.22(1.01-1.47)		1.16(0.69-1.95)	
	Other	98 (95.1)	1.35(0.86-2.12)		0.56(0.24-1.32)	
**Fathers education**	No education	2326 (96.1)	1	0.150	1	**<0.001**
	Primary	655 (97.1)	0.95(0.78-1.16)		2.01(1.24-3.26)	
	Secondary	2063 (97.3)	1.26(1.09-1.45)		2.36(1.69-3.31)	
	Tertiary	1827 (97.8)	0.88(0.75-1.04)		3.46(2.29-5.22)	
	Quranic/Arabic	2033 (96.9)	0.98(0.85-1.14)		2.49(1.77-3.50)	
	Don`t know	1077 (97.0)	1.14(0.95-1.36)		2.12(1.39-3.23)	
**Mothers education**	No education	2941 (97.3)	1	**<0.001**	1	**<0.001**
	Primary	1137 (97.3)	1.38(1.18-1.61)		0.94(0.62-1.43)	
	Secondary	2176 (98.0)	0.92(0.81-1.04)		0.75(0.52-1.08)	
	Tertiary	1009 (98.5)	0.91(0.77-1.07)		0.41(0.26-0.65)	
	Quranic/Arabic	1676 (94.5)	0.95(0.83-1.08)		0.30(0.21-0.42)	
	Don`t know	1040 (96.2)	0.83(0.71-0.98)		0.45(0.30-0.69)	
**Living with parents**	No	7988 (96.8)	1	**0.001**	1	**0.008**
	Yes	1987 (97.9)	0.83(0.74-0.93)		0.66(0.49-0.89)	
**Smoking Status**	Non smokers	8309 (97.0)	1	**<0.001**	1	0.250
	Ever smokers	1671 (97.2)	1.63(1.31-2.03)		1.46(0.76-2.82)	
**Smoking at home allowed**	No	7060 (96.7)	1	**<0.001**	1	0.524
	Sometimes	1070 (98.6)	2.29(1.99-2.64)		0.88(0.62-1.25)	
	Yes	1849 (97.0)	2.73(2.43-3.07)		0.86(0.65-1.14)	
**Family smoking**	None	7112 (96.5)	1	**<0.001**	1	0.060
	Mother	270 (98.5)	1.66(1.27-2.18)		2.64(0.95-7.29)	
	Father	1177 (98.1)	3.16(2.74-3.65)		0.75(0.54-1.04)	
	Sibling	702 (97.7)	1.76(1.49-2.08)		1.14(0.72-1.81)	
	Others	716 (98.2)	2.09(1.77-2.47)		1.33(0.80-2.21)	
**Number of friends who smoke**	None	6563 (96.6)	1	**<0.001**	1	0.062
	one	653 (97.0)	1.89(1.58-2.26)		1.11(0.71-1.73)	
	Two	344 (96.6)	1.94(1.52-2.47)		1.14(0.63-2.08)	
	Three or more	743(97.5)	1.53(1.28-1.82)		1.39(0.85-2.25)	
	Not sure	1670 (98.2)	1.13(1.00-1.28)		1.63(1.15-2.30)	
**Sent to buy cigarettes**	**No**	5700 (98.0)	1	**<0.001**	1	**0.001**
	**Yes**	4271 (95.7)	1.98(1.80-2.18)		1.42(1.14-1.77)	
**Ban at enclosed public places**	No	5638 (97.8)	1	**<0.001**	1	0.191
	Yes	4336 (95.9)	1.22(1.09-1.37)		1.19(0.91-1.55)	
**Ban at outdoor public places**	No	5713 (97.6)	1	**0.024**	1	0.760
	Yes	4259 (96.2)	0.88(0.78-0.98)		1.04(0.80-1.35)	

SHS, second-hand smoke
UBS, upper basics schools
SSS, senior secondary schools

For peer review only - http://bmjopen.bmj.com/site/about/guidelines.xhtml

For peer review only

DISCUSSION

This is the first study to provide detailed data on exposure to SHS in a nationally representative sample of adolescent school students in The Gambia. We found a very high level of self-reported exposure to SHS among students, and, contrary to expectation, found that while around two in five respondents reported SHS exposure in the home, a large majority of young people reported exposure in public places. Older students in our sample were generally more likely to be exposed to SHS, as were children under the age of 15. Older students and girls were significantly more like to be exposed to SHS at home compared to boys. Students in our sample were also more likely to be exposed to SHS if their family members or friends smoked, if they attended UBS or grant-aided schools, smoking was allowed in the home and among those who were not Muslim. Exposure to SHS at home and outside the home were also associated with parental educational level, though in opposite ways; higher maternal and lowest paternal levels of education were associated with lower exposure. Students who were sent to purchase cigarettes for others were also more likely to be exposed to SHS. Awareness of the harm to health of SHS exposure was low; with more than a quarter of students reporting that exposure was probably or definitely not harmful. However most students supported a smoking ban in both enclosed and outdoor public places. Our study has some limitations. This was a cross-sectional study and we used a self-administered questionnaire to measure exposure to SHS: students may have under or over reported the answers. However, students self-reports of exposure to SHS has been reported to be highly consistent with urinary cotinine level measurement (11). Our sampling method ensured that the population selected was likely to be highly representative of young people in The Gambia, while we recognised that this limits the generality of our findings to young people not in school. Data from the Ministry of Basic and Secondary Education (MoBSE) indicated gross enrolment rates of 68.1% and 41.2% for UBS and SSS respectively and the

For peer review only - http://bmjopen.bmj.com/site/about/guidelines.xhtml

universal primary and secondary education initiative which have seen greater number of young people go to school in the Gambia (12). Furthermore, the present study has a number of strengths that include a large sample size and high response rate among those interviewed, which also supports the robustness of the study findings. Additionally, the study addressed SHS exposure both at home and outside the household.

Previous studies of smoking and exposure to SHS among students in The Gambia are limited, the most recent and widely quoted being the 2008 GYTS survey. The current study estimated a lower overall prevalence of exposure to SHS than ours, but this could well reflect the restricted local sampling frame used in the GYTS (9). The high prevalence of exposure to SHS is consistent with earlier studies in the Gambia and other countries in Africa (13, 14). In the Gambia, the Public Smoking Act, which bans smoking in all public places, came into effect in 1998. However, our observation that exposure to SHS remains high, and may even have increased since the 2008 GYTS, suggests that efforts are still needed to enhance the enforcement of this law, particularly since public places were the most frequent source of exposure to SHS among young people in The Gambia. Beyond the direct health benefit of smoke-free policies, implementing smoke-free laws, especially in public places, has been shown to change the public acceptance of smoking by the general population (15, 16). Most countries in the African region still have weak or even non-existent smoke free laws and compliance with smoke–free laws varies extensively (6). Furthermore, enforcement of smoke free polices in most African countries have been identified as a major challenge (17).

Similar to previous findings (18), our study also showed that more than half of the students are supportive of polices that ban public smoking; however many are unaware of the harmful effects of exposure to SHS. Adolescence awareness of the harmful health effects of SHS has been shown to be associated with a reduced risk of exposure to SHS (19, 20), and suggests that improved education on the risks of SHS could lead to reductions in exposure.

For peer review only - http://bmjopen.bmj.com/site/about/guidelines.xhtml

We found that older students were more likely to be exposed to SHS both outside the home and inside the home; this is consistent with findings in previous studies among students (21, 22). Older students have more opportunities to be outside the home in public places where smoking is more likely to happen. Our finding that parents' educational level, living with parents and being sent to purchase cigarette for others were significant determinants of exposure to SHS in public places is consistent with previous studies (18, 23, 24), and probably arises from the fact that these characteristics all identify contact with others who smoke.

All of the participant schools in this study reported that they had implemented a comprehensive smoke–free campus policy, yet more than a quarter of students reported SHS exposure at school. These findings, which are consistent with previous reports of significant exposure to SHS at school (25-27) suggest that enforcement of school-based tobacco control measures needs to be strengthened. Studies have shown that in schools with comprehensive policies and high compliance, students are much less likely to report exposure, and report lower intentions to smoke in the future (28).

Our results showed that parents' educational level, family or friends' smoking status, living with parents, home smoking rules and being sent to purchase cigarette for others were significant determinants of exposure to SHS in the home and is consistent with previous studies (18, 21-24). Furthermore it has also been shown that non-smokers exposed to SHS at home are more likely to be susceptible to initiating smoking than those not exposed (29). Educating parents about the harmful effects of smoking and exposure to SHS could be one of the effective ways to protect young people at home. This will help to protect children, help parents who smoke to quit and discourage others from smoking in their homes.

This study has shown that exposure to SHS is very high among students and that despite smoke-free laws, protection against SHS exposure in public places in the Gambia is still inadequate. There is an urgent need to advocate for interventions to reduce the current level of exposure to SHS and minimise further exposure among students. This underscores the

For peer review only - http://bmjopen.bmj.com/site/about/guidelines.xhtml

need to develop comprehensive smoke-free laws and strictly enforce these laws in all environments. Further research is required to determine whether this is a problem among students alone, or reflects a wider pattern of exposure to SHS among the general population.

Acknowledgements: We thank all the students who participated and the teachers for helping in the coordination; and also all the

For peer review only

For peer review only - http://bmjopen.bmj.com/site/about/guidelines.xhtml

Regional Education Directors and the Ministry of Basic and Secondary Education. We profoundly recognise the contributions of the field workers

and staff of the National Public Health Laboratory (NPHL) . We also acknowledged the support of the Tobacco Control Unit of the Ministry of Health and Social Welfare.

Competing interests: None.

Contributors: IJ, study design, data collection, analysis and drafting of manuscript; TL and JB, study design and revision of manuscript.

Funding: This work was supported by the Medical Research Council [grant number MR/K023195/1] through the UK Centre for Tobacco and Alcohol Studies; and a scholarship funding from the Islamic Development Bank (IDB).

Ethics approval: This research project was reviewed and approved by The Gambia Government/Medical Research Council (MRC) Joint Ethics Committee in The Gambia, and the Research Ethics Committee of the Faculty of Medicine and Health Sciences, University of Nottingham, UK.

Provenance and peer review: externally peer reviewed**.**

Data sharing statement: No additional data is available.

For peer review only

REFERENCES

1. Oberg M, Jaakkola MS, Woodward A, Peruga A, Pruss-Ustun A. Worldwide burden of disease from exposure to second-hand smoke: a retrospective analysis of data from 192 countries. Lancet. 2011;377(9760):139-46.
2. WHO. Worldwide burden of disease from exposure to second-hand smoke. http://wwwwhoint/quantifying_ehimpacts/publications/shsarticle2010/en/ (accessed 20June 2017).
3. Center for Disease Control and Prevention. The Health Consequences of Involuntary Exposure to Tobacco Smoke; A Report of the Surgeon General. 2006.
4. WHO. WHO global report on mortality attributed to toobacco. World Health Organization. 2012.
5. WHO Report on the Global Tobacco Epidemic. Raising tax on tobacco; mpower. 2015.
6. WHO Report on the Global Tobacco Epidemic: Monitoring tobacco use and prevention polices. 2017.(accessed December 2017)
7. World Bank World Development Indicator Database 2015http://dataworldbankorg/country/gambia-the (accessed March 2017).
8. Welfare MoHS. Prohibation of Smoking (Public Places) Act 1998.
9. Manneh E. The Gambia. A Global Youth Tobacco Survey (GYTS) Country Report (2008)2008 08/11/2016. Available from: http://apps.who.int/fctc/reporting/party_reports/gambia_annex2_gyts_2008_final_report.pdf.
10. Jallow IK, Britton J, Langley T. Prevalence and determinants of tobacco use among young people in The Gambia. BMJ Global Health, in press. 2017.
11. Ekerbicer HC, Celik M, Guler E, Davutoglu M, Kilinc M. Evaluating environmental tobacco smoke exposure in a group of Turkish primary school students and developing intervention methods for prevention. BMC Public Health. 2007;7:202.
12. The Ministry of Basic and Secondary Education & Gambia National Commission for UNESCO. The Gambia National Education For All Review Report 2014. http://unesdoc.unesco.org/images/0023/002314/231425e.pdf.
13. Lee KA, Palipudi KM, English LM, Ramanandraibe N, Asma S. Secondhand smoke exposure and susceptibility to initiating cigarette smoking among never-smoking students in selected African countries: Findings from the Global Youth Tobacco Survey. Prev Med. 2016;91S:S2-S8.
14. Owusu D, Mamudu HM, John RM, Ibrahim A, Ouma AE, Veeranki SP. Never-Smoking Adolescents' Exposure to Secondhand Smoke in Africa. Am J Prev Med. 2016;51(6):983-98.
15. Husain MJ, English LM, Ramanandraibe N. An overview of tobacco control and prevention policy status in Africa. Prev Med. 2016;91s:S16-s22.
16. Almutairi KM. Prevalence of tobacco use and exposure to environmental tobacco smoke among saudi medical students in Riyadh, Saudi Arabia. J Community Health. 2014;39(4):668-73.
17. Tumwine J. Implementation of the framework convention on tobacco control in Africa: current status of legislation. Int J Environ Res Public Health. 2011;8(11):4312-31.

For peer review only - http://bmjopen.bmj.com/site/about/guidelines.xhtml

18. Peltzer K. Determinants of exposure to second-hand tobacco smoke (SHS) among current non-smoking in-school adolescents (aged 11-18 years) in South Africa: results from the 2008 GYTS study. Int J Environ Res Public Health. 2011;8(9):3553-61.
19. Rudatsikira E, Siziya S, Dondog J, Muula AS. Prevalence and correlates of environmental tobacco smoke exposure among adolescents in Mongolia. Indian J Pediatr. 2007;74(12):1089-93.
20. Raute LJ, Pednekar MS, Mistry R, Gupta PC, Pimple SA, Shastri SS. Determinants of exposure to second-hand smoke at home and outside the home among students aged 11-17 years: results from the Mumbai Student Tobacco Survey 2010. Indian J Cancer. 2012;49(4):419-24.
21. Rachiotis G, Siziya S, Muula AS, Rudatsikira E, Papastergiou P, Hadjichristodoulou C. Determinants of exposure to Environmental Tobacco Smoke (ETS) among non smoking adolescents (aged 11-17 years old) in Greece: results from the 2004-2005 GYTS Study. Int J Environ Res Public Health. 2010;7(1):284-90.
22. Jordaan ER, Ehrlich RI, Potter P. Environmental tobacco smoke exposure in children: household and community determinants. Arch Environ Health. 1999;54(5):319-27.
23. Lee K, Hahn EJ, Riker CA, Hoehne A, White A, Greenwell D, et al. Secondhand smoke exposure in a rural high school. The Journal of School Nursing. 2007;23(4):222-8.
24. Cartmell KB, Miner C, Carpenter MJ, Vitoc CS, Biggers S, Onicescu G, et al. Secondhand smoke exposure in young people and parental rules against smoking at home and in the car. Public Health Rep. 2011;126(4):575-82.
25. Gonzales M, Malcoe LH, Kegler MC, Espinoza J. Prevalence and predictors of home and automobile smoking bans and child environmental tobacco smoke exposure: a cross-sectional study of U.S.- and Mexico-born Hispanic women with young children. BMC Public Health. 2006;6:265.
26. Fallin A, Roditis M, Glantz SA. Association of campus tobacco policies with secondhand smoke exposure, intention to smoke on campus, and attitudes about outdoor smoking restrictions. Am J Public Health. 2015;105(6):1098-100.
27. Farrelly MC, Evans WN, Sfekas AE. The impact of workplace smoking bans: results from a national survey. Tob Control. 1999;8(3):272-7.
28. Stillman F, Navas-Acien A, Ma J, Ma S, Avila-Tang E, Breysse P, et al. Second-hand tobacco smoke in public places in urban and rural China. Tobacco control. 2007;16(4):229-34.
29. Wakefield M, Banham D, Martin J, Ruffin R, McCaul K, Badcock N. Restrictions on smoking at home and urinary cotinine levels among children with asthma. Am J Prev Med. 2000;19(3):188-92.

For peer review only

For peer review only - http://bmjopen.bmj.com/site/about/guidelines.xhtml

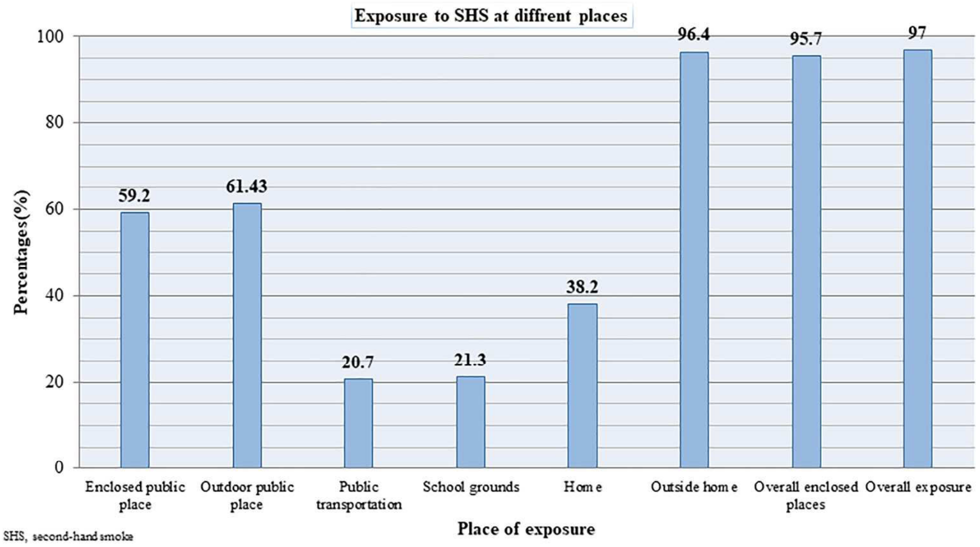

Figure 1 Exposure to SHS at different locations

228x132mm (300 x 300 DPI)

STROBE Statement—Checklist of items that should be included in reports of ***cross-sectional studies***

	Item No	Recommendation	Reported on page #
**Title and abstract**	1	(*a*) Indicate the study's design with a commonly used term in the title or the abstract	1
		(*b*) Provide in the abstract an informative and balanced summary of what was done and what was found	2
**Introduction**			
Background/rationale	2	Explain the scientific background and rationale for the investigation being reported	4
Objectives	3	State specific objectives, including any prespecified hypotheses	4
**Methods**			
Study design	4	Present key elements of study design early in the paper	5
Setting	5	Describe the setting, locations, and relevant dates, including periods of recruitment, exposure, follow-up, and data collection	5
Participants	6	(*a*) Give the eligibility criteria, and the sources and methods of selection of participants	5
Variables	7	Clearly define all outcomes, exposures, predictors, potential confounders, and effect modifiers. Give diagnostic criteria, if applicable	6
Data sources/ measurement	8*	For each variable of interest, give sources of data and details of methods of assessment (measurement). Describe comparability of assessment methods if there is more than one group	6
Bias	9	Describe any efforts to address potential sources of bias	6
Study size	10	Explain how the study size was arrived at	5
Quantitative variables	11	Explain how quantitative variables were handled in the analyses. If applicable, describe which groupings were chosen and why	6
Statistical methods	12	(*a*) Describe all statistical methods, including those used to control for confounding	6
		(*b*) Describe any methods used to examine subgroups and interactions	6
		(*c*) Explain how missing data were addressed	6
		(*d*) If applicable, describe analytical methods taking account of sampling strategy	NA
		(*e*) Describe any sensitivity analyses	NA
**Results**			
Participants	13*	(a) Report numbers of individuals at each stage of study—eg numbers potentially eligible, examined for eligibility, confirmed eligible, included in the study, completing follow-up, and analysed	7
		(b) Give reasons for non-participation at each stage	7
		(c) Consider use of a flow diagram	7
Descriptive data	14*	(a) Give characteristics of study participants (eg demographic, clinical, social) and information on exposures and potential confounders	8
		(b) Indicate number of participants with missing data for each variable of interest	9
Outcome data	15*	Report numbers of outcome events or summary measures	10 & 11
Main results	16	(*a*) Give unadjusted estimates and, if applicable, confounder-adjusted estimates and their precision (eg, 95% confidence interval). Make clear	13

For peer review only - http://bmjopen.bmj.com/site/about/guidelines.xhtml

		which confounders were adjusted for and why they were included	
		(*b*) Report category boundaries when continuous variables were categorized	13
		(*c*) If relevant, consider translating estimates of relative risk into absolute risk for a meaningful time period	NA
Other analyses	17	Report other analyses done—eg analyses of subgroups and interactions, and sensitivity analyses	NA
**Discussion**			
Key results	18	Summarise key results with reference to study objectives	14
Limitations	19	Discuss limitations of the study, taking into account sources of potential bias or imprecision. Discuss both direction and magnitude of any potential bias	14
Interpretation	20	Give a cautious overall interpretation of results considering objectives, limitations, multiplicity of analyses, results from similar studies, and other relevant evidence	15-16
Generalisability	21	Discuss the generalisability (external validity) of the study results	17
**Other information**			
Funding	22	Give the source of funding and the role of the funders for the present study and, if applicable, for the original study on which the present article is based	17

*Give information separately for exposed and unexposed groups.

Note: An Explanation and Elaboration article discusses each checklist item and gives methodological background and published examples of transparent reporting. The STROBE checklist is best used in conjunction with this article (freely available on the Web sites of PLoS Medicine at http://www.plosmedicine.org/, Annals of Internal Medicine at http://www.annals.org/, and Epidemiology at http://www.epidem.com/). Information on the STROBE Initiative is available at www.strobe-statement.org.

For peer review only

For peer review only - http://bmjopen.bmj.com/site/about/guidelines.xhtml
